# A performance evaluation of drug response prediction models for individual drugs

**DOI:** 10.1038/s41598-023-39179-2

**Published:** 2023-07-24

**Authors:** Aron Park, Yeeun Lee, Seungyoon Nam

**Affiliations:** 1 Department of Health Sciences and Technology, Gachon Advanced Institute for Health Sciences and Technology (GAIHST), Gachon University, Incheon, 21999 Republic of Korea; 2 Department of Genome Medicine and Science, AI Convergence Center for Medical Science, Gachon University Gil Medical Center, Gachon University College of Medicine, Incheon, 21565 Republic of Korea

**Keywords:** Computational biology and bioinformatics, Machine learning

## Abstract

Drug response prediction is important to establish personalized medicine for cancer therapy. Model construction for predicting drug response (i.e., cell viability half-maximal inhibitory concentration [IC_50_]) of an individual drug by inputting pharmacogenomics in disease models remains critical. Machine learning (ML) has been predominantly applied for prediction, despite the advent of deep learning (DL). Moreover, whether DL or traditional ML models are superior for predicting cell viability IC_50s_ has to be established. Herein, we constructed ML and DL drug response prediction models for 24 individual drugs and compared the performance of the models by employing gene expression and mutation profiles of cancer cell lines as input. We observed no significant difference in drug response prediction performance between DL and ML models for 24 drugs [root mean squared error (RMSE) ranging from 0.284 to 3.563 for DL and from 0.274 to 2.697 for ML; R^2^ ranging from −7.405 to 0.331 for DL and from −8.113 to 0.470 for ML]. Among the 24 individual drugs, the ridge model of panobinostat exhibited the best performance (R^2^ 0.470 and RMSE 0.623). Thus, we selected the ridge model of panobinostat for further application of explainable artificial intelligence (XAI). Using XAI, we further identified important genomic features for panobinostat response prediction in the ridge model, suggesting the genomic features of 22 genes. Based on our findings, results for an individual drug employing both DL and ML models were comparable. Our study confirms the applicability of drug response prediction models for individual drugs.

## Introduction

Drug response prediction is crucial to identify appropriate therapies for patients with cancer. However, extensive clinical trials to predict drug responses in diverse cancers remain impracticable, accompanied by unaffordable costs^1–4^. Consequently, cancer cell lines have been established as disease models to overcome these limitations, resulting in the emergence of drug response research for developing pharmacogenomic databases using cancer cell lines^1^. Large-scaled pharmacogenomic databases for cancer cell lines, such as Cancer Cell Line Encyclopedia (CCLE) and Genomics of Drug Sensitivity in Cancer (GDSC), have enabled the construction of drug response prediction models using artificial intelligence^2,3^.

To date, several studies have attempted to construct drug response prediction models using these pharmacogenomic databases as training data^5–19^. In these studies, to predict the drug response (i.e., cell viability half-maximal inhibitory concentration [IC_50_]) of various individual drugs on a cancer cell line, the genomic profile (e.g., mutations and gene expression profiles) of a cancer cell line was used as input data^6–14^. However, these studies were primarily based on machine-learning (ML) method. Importantly, the superiority of ML or deep learning (DL) models for predicting responses of an individual drug is yet to be determined.

Furthermore, genomics features affecting the values predicted in the drug response prediction model need to be derived. Consequently, explainable artificial intelligence (XAI) technique^20^ was introduced in predicting models. The XAI technique enables the introduction of important features that affect the predicted values in the model. However, few studies have explored the application of XAI for constructing drug response prediction models, especially considering data from patients with cancer. Hence, it is critical to establish a drug response prediction model for individual drugs and identify important genomic features using XAI.

To address these limitations, we constructed two datasets by combining the drug response data from CCLE and gene expression and mutation profiles from CCLE and GDSC, respectively. Next, we established two input settings for the drug response prediction models for 24 individual drugs (a model for each drug). We compared the prediction performance of DL and ML models in the two input settings. Additionally, we identified the major genomic features affecting drug sensitivity by applying XAI to the best model based on the performance comparison.

## Results

### Overview

**Figure 1:**
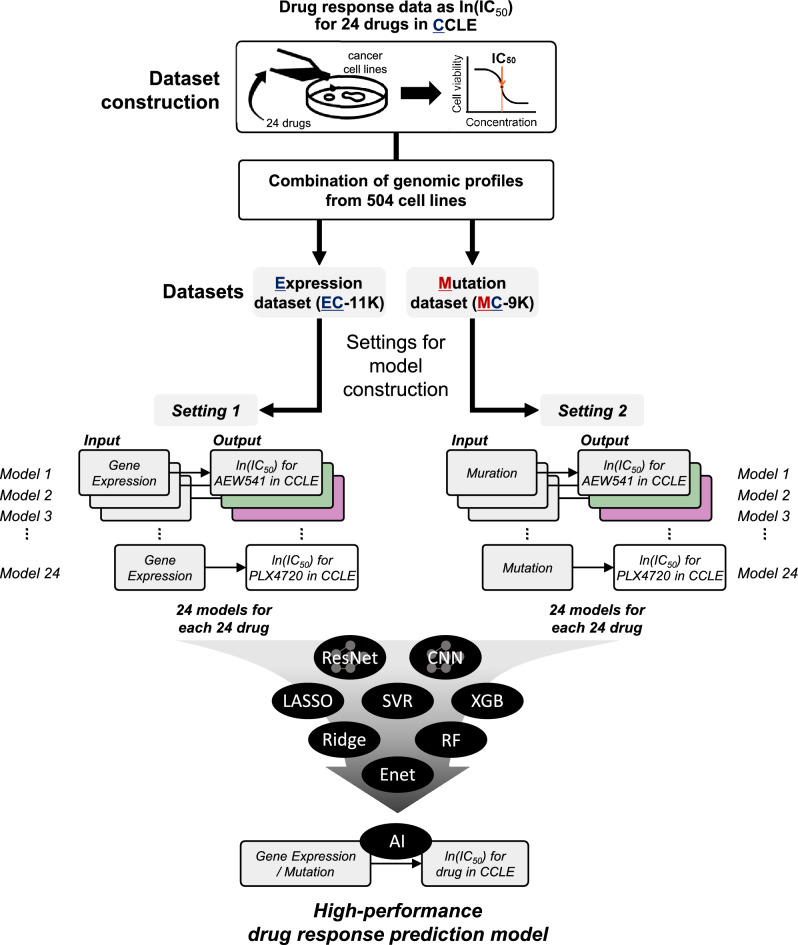
Dataset and model description. Considering the drug response data as learning data for our prediction target (ln[IC_50_] values), we combined two types of data, including genomic information (gene expression and mutation profiles). This yielded expression (EC-11K) and mutation (MC-9K) datasets for the drug-response prediction model. We set two input settings to construct drug response, prediction models. Settings 1 and 2 handle gene expression profiles (mutation profiles for setting 2) to predict ln(IC_50_) values for an individual drug in one model, such that settings 1 and 2 had a total of 24 models (for prediction of drug response for 24 drugs). We have used three abbreviations: E (*e*xpression), M (*m*utation), and C (drug response of *C*CLE cell lines, ln[IC_50_]). CCLE, Cancer Cell Line Encyclopedia.

Herein, we first constructed two datasets, a gene expression dataset and a mutation dataset, to establish a drug response prediction model. Each dataset was named to describe the data (Fig. 1) and the number of cases in Supplementary Table S1. For example, one combination was named EC-11K, representing a combined dataset of gene *e*xpression data (denoted as “E”), and ln(IC_50_)s as drug response measurements from the *C*CLE (denoted as “C”), consisting of a total of ~ *11,000* (11K) cases. The other dataset was named MC-9K, including *m*utation statuses (denoted as “M”) and ln(IC_50_)s from the *C*CLE, comprising ~ *9000* (9K) cases (Supplementary Table S1).

**Figure 2:**
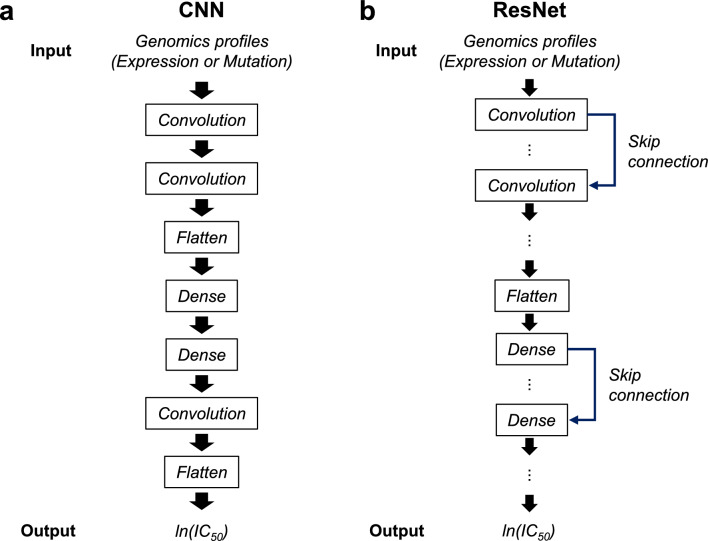
Conceptual architecture of ResNet and CNN. (**a**) ResNet features usage of skip connection (**b**) CNN architecture adopted from CDRscan, except for the convolutional neural network layers for a drug.

Subsequently, we constructed two input settings (settings 1 and 2) to predict the response of an individual drug using two types of datasets. Setting 1 was the construction of 24 drug response prediction models for 24 individual drugs, considering the gene expression dataset (EC-11K) as the input. Setting 2 employed the same approach as in setting 1 using the mutation dataset (MC-9K) (Fig. 1, Supplementary Fig. S1, Supplementary Tables S2, and S3). For each setting, we constructed DL models, using architecture of convolutional neural network (CNN) (Fig. 2a) and ResNet (Fig. 2b), and ML models [lasso, ridge, support vector regression (SVR), random forest (RF), extreme gradient boosting (XGBoost), and ElasticNet (Enet)] to predict ln(IC50) values. We adopted ‘CDRscan master’ model (henceforth, CDRscan) as CNN^15^.

### Setting 1: model construction for individual drugs, considering *e*xpression profiles (EC-11K) as input for prediction of ln(IC_50_)s

Under setting 1, with gene expression profiles as the input, we constructed an ln(IC_50_) drug responsiveness prediction model for individual drugs (Supplementary Fig. S1a). In the aforementioned instances, a few hundred ln(IC_50_) measurements were available for individual drugs in diverse cancer cell lines (Supplementary Table S2). For each method (CNN, ResNet, lasso, ridge, SVR, RF, and XGBoost, Enet), we constructed drug response prediction models using gene expression profiles for individual drugs, using the training set from EC-11K (Supplementary Table S2).

Using test sets, the prediction performances of 24 drugs using DL and ML models were described using the root mean squared error (RMSE) and R-squared value (R^2^) (Supplementary Tables S4, S5, S6, S7, S8, S9, S10, and S11; Supplementary Figs. S2, S3, S4, S5, S6, S7, S8, and S9).

**Figure 3:**
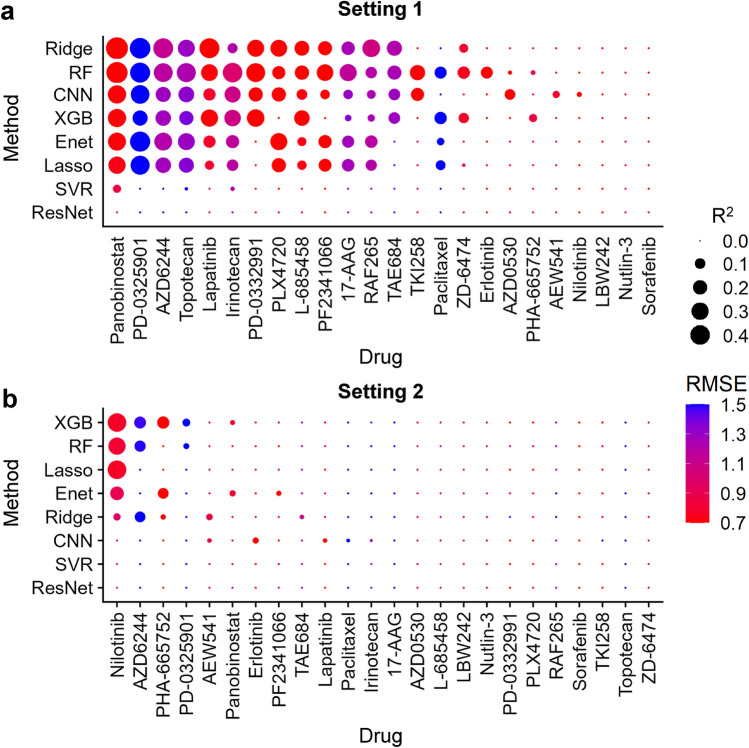
Integrated heatmap with dot plot for performance comparisons in settings 1 and 2. The R^2^ value is depicted as a size of the circle. Red and blue colors indicate RMSE values. The model with higher R^2^ and lower RMSE value is considered to have good performance. Note that R^2^ with a 0.0 value indicates that the R^2^ values of each model exhibit negative values or zero. (**a**) In setting 1, the ridge model for panobinostat shows the best performance among other models (**b**) In setting 2, no model outperformed the ridge model for panobinostat in setting 1. *R*^*2*^ R-squared value, *RMSE* root mean squared error.

Accordingly, the ridge prediction model for panobinostat showed the best performance (R^2^: 0.470 and RMSE: 0.623) when compared with all models for other drugs (Fig. 3a, Supplementary Figure S3, and Supplementary Table S5). Notably, we detected no significant difference in drug response prediction performance between the DL and ML models for 24 drugs (RMSE ranging from 0.284 to 3.563 for DL and from 0.274 to 2.564 for ML; R^2^ ranging from −2.763 to 0.331 for DL and from −8.113 to 0.470 for ML).

We also inspected whether fine-tuning (i.e., feature selection) improved the performances of CNN and ridge for panobinostat. For the feature section, lasso was used in the training set. The numbers of features selected by lasso were 2000, 4000, 6000, 8000, 10,000, 12,000, 14,000, 16,000, and 18,988 (i.e., whole features). Given the features, we trained CNN and ridge, and measured the performances of the two models. As a result, the best prediction model in the two models was the ridge model using whole features (Supplementary Fig. S10). We attempted two more feature engineering techniques [i.e., non-linear feature generation^21^ and feature selection by ‘lasso with lars’ (least angle regression and shrinkage)^22^] using the Autofeat library of Python. As a result, the R^2^ values for the two feature engineering techniques were 0.218 and 0.409, respectively, which were not improved.

### Setting 2: model construction for individual drugs, taking *m*utation profiles (MC-9K) as input for prediction of ln(IC50)s

For setting 2, similar to setting 1, 24 individual drug prediction models were constructed using mutation profiles using the training set from MC-9K (Supplementary Fig. S1b and Supplementary Table S3). Comparing the performance using test sets, all models failed to display strong positive correlations between predicted and actual ln(IC_50_) values for any drug (Fig. 3b; Supplementary Figs. S11, S12, S13, S14, S15, S16, S17, and S18; Supplementary Tables S12, S13, S14, S15, S16, S17, S18 and S19).

### Application of ridge for panobinostat from setting 1 to gastric cancer (GC) cell lines and patient datasets

As mentioned in setting 1, the ridge model for panobinostat exhibited better performance than all the models for other drugs. Panobinostat is a histone deacetylase (HDAC) inhibitor. HDAC2 was overexpressed in gastric cancer (GC) cell lines to utilize panobinostat for GC treatment^23^.

**Figure 4:**
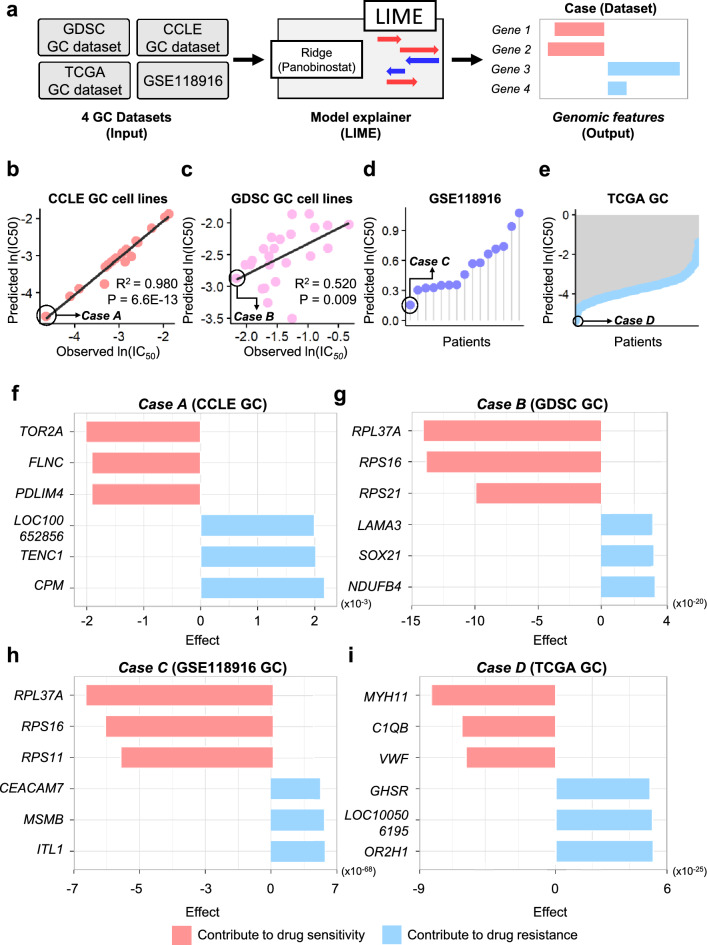
Application of the ridge model for panobinostat in setting 1 to the GC cell line (CCLE and GDSC) and patient datasets (GSE118916 and TCGA). (**a**) Description of XAI application. (**b**) CCLE and (**c**) GDSC GC cell lines show a good correlation between predicted and observed ln(IC_50_) values. (**d**) GSE118916 and (**e**) TCGA datasets for patients with GC show a broad distribution of predicted ln(IC_50_) values. Furthermore, we selected a sensitive case for panobinostat in each GC dataset, and cases A through D are indicated in the circle. For case A and B, the predicted IC_50_ values of the two cases agree with their observed IC_50_ values. Considering case C and D, their datasets did not have patient information on the response, and the prediction cannot be compared. Considering prediction, the four cases are sensitive to the panobinostat when compared with the other samples.** (f–i)** To identify major genes affecting drug response, we performed XAI analysis with the four cases (case A through D). The six genes affecting drug sensitivity (red bar) and drug resistance (blue bar) were detected in **(f)** case A, **(g)** case B, **(h)** case C, and **(i)** case D, respectively. *CCLE* Cancer Cell Line Encyclopedia, *GDSC* genomics of drug sensitivity in cancer, *GC* gastric cancer, *TCGA* The Cancer Genome Atlas, *XAI* explainable artificial intelligence.

In addition, given that GC tumors are highly heterogeneous^24^, we applied the ridge model of panobinostat to GC cell lines and patients with GC. We obtained four datasets of gene expression profiles of GC from CCLE^2^, GDSC^3,25^, GSE118916^26^, and patients with GC (n = 450) from The Cancer Genome Atlas (TCGA)^27^. We then entered the gene expression status vectors from the four datasets into the ridge model. For each dataset, we obtained a predicted ln(IC_50_) value for each cell line (or patient) for panobinostat (Fig. 4a).

Using ln(IC_50_) prediction values from each dataset, we first compared the observed ln(IC_50_)s of the drugs in CCLE and GDSC GC cell lines to predict the drug ln(IC_50_)s. Both CCLE and GDSC GC cell lines presented a strong positive correlation (R^2^ for CCLE GC: 0.980 and R^2^ for GDSC GC: 0.520) between predicted and observed ln(IC_50_) values (Fig. 4b, c).

Second, we attempted to predict ln(IC_50_) values from patients with GC (GSE118916 and TCGA GC). In particular, the patient dataset yielded results revealing a broad distribution, which indicated that the drug response of panobinostat differed from the gene expression profile of each patient, thereby implying that the ridge model considers the heterogeneity of GC tumors^27^ (Fig. 4d, e).

### Inspection of major genomic features affecting drug response prediction using *l*ocal *i*nterpretable *m*odel-agnostic *e*xplanation (LIME) analysis

We investigated ln(IC_50_) predictions by inputting each of the four GC datasets into the ridge (panobinostat) model in setting 1. We confirmed that the drug response of each GC cell line (or patient) to panobinostat was predicted distinctly according to the gene expression vector. Accordingly, by utilizing an XAI approach such as LIME, we extracted significant genomic features in the ridge model of panobinostat in setting 1. Subsequently, we selected a sensitive case for panobinostat in each dataset, with case A for the CCLE GC dataset, case B for the GDSC GC dataset, case C for the GSE118916 GC dataset, and case D for the TCGA GC dataset, denoted in Fig. 4b–e, respectively. Given that the two datasets (GDSC and CCLE) contained actual IC_50s_ derived from cell viability assays, the predicted IC_50s_ of the two cases (cases A and B) were compared with their observed IC_50s_ to establish confirmation between the prediction and the observation. Furthermore, the four cases (A, B, C, and D) were predicted to be more sensitive to panobinostat when compared with that of the other samples. Then, by applying LIME to the ridge model, we inspected the top three explainable genes affecting drug response and the top three genes affecting drug response in each case (cases A through D).

The top three genes that most affected drug response were *TOR2A*, *FLNC*, and *PDLIM4* in case A; *RPL37A*, *RPS16*, and *RPS21* in case B; *RPL37A*, *RPS16*, and *RPS11* in case C; and *MYH11*, *C1QB*, and VWF in case D (red bars in Fig. 4f–i). Conversely, the top three genes impacting drug resistance were *LOC100652856*, *TENC1*, and *CPM* in case A; *LAMA3*, *SOX21*, and *NDUFB4* in case B; *CEACAM7*, *MSMB*, and *ITLN1* in case C; *GHSR*, *LOC100506195*, and *OR2H1* in case D (blue bars in Fig. 4f–i).

Next, we inspected whether application of XAI to the ridge (panobinostat) model in setting 1 could reveal novel features other than the features selected by the ridge model in setting 1. For the purpose, we compared the features by the XAI with those by the ridge (panobinostat) model. As a result, the gene features selected by XAI were found to overlap with only three genes of the gene features selected in the panobinostat ridge model. It indicates that XAI selected novel gene features (Supplementary Fig. S19).

Since XAI is usually applied to DL, we applied LIME to CNN (i.e., CDRscan) in setting 1. For case D, the same important features were selected in both the panobinostat ridge model and CNN. For cases A, B, and C, no overlapped selected features with the panobinostat ridge model and CNN were revealed (Supplementary Fig. S20).

## Discussion

Using diverse input settings, we compared the performance of ML and DL models in predicting IC_50_ cell viability values as drug responses based on pharmacogenomic databases by employing gene expression and mutation profiles.

Following this scheme, we also constructed AI and ML models, for individual drug species, for the EC-11K and MC-9K datasets (settings 1 and 2). Considering visual inspection (Supplementary Figs. S2, S3, S4, S5, S6, S7, S8, S9, S11, S12, S13, S14, S15, S16, S17, and S18), R^2^, and RMSE (Supplementary Tables S4, S5, S6, S7, S8, S9, S10, S11, S12, S13, S14, S15, S16, S17, S18, and S19), we noted that DL and ML models did not exhibit substantial differences in drug response prediction performance for 24 drugs (RMSE ranging from 0.284 to 3.563 for DL and from 0.274 to 2.697 for ML; R^2^ ranging from -7.405 to 0.331 for DL and from -8.113 to 0.470 to ML). Notably, the panobinostat ridge prediction model outperformed other models (Fig. 3a).

On applying the ridge (panobinostat) model in setting 1 to patients with GC, CCLE and GDSC GC revealed a positive correlation between the predicted and observed ln(IC_50_)s (Fig. 4b, c). However, we failed to detect any correlation between predicted and observed drug responses in GSE118916 and TCGA GC, given the lack of drug response data for panobinostat. Nevertheless, we observed that patients with GC exhibited a heterogeneous drug response to panobinostat (Fig. 4d, e).

In addition, we performed XAI analysis using LIME to identify the major genes that affect drug responsiveness prediction (drug sensitivity and resistance) in patients with GC (Fig. 4f–i). Using XAI, we confirmed that the genes affecting drug response and resistance in each case were related to the onset of GC and other cancers^4,7,15,26,28–56^. Interestingly, among genes affecting drug response prediction, *CPM* was found to contribute to chemoresistance in GC^49^, whereas *RPL37A* was a potential biomarker for response to neoadjuvant chemotherapy (NCT) against non-metastatic locally advanced breast carcinoma (LABC)^41^. Furthermore, we observed that the upregulation of *MYH11* inhibited tumor growth in GC^45^, and genes encoding ribosomal proteins (*RPL16*, *RPS11*, and *RPS21*) were related to ulcerative colitis and GC. Accordingly, XAI analysis can reveal important genes that affect drug responses based on the genomic profile of each patient. Additionally, it can be used as a personalized medical strategy to overcome the heterogeneity observed in patients with cancer.

The limitations of the present study need to be addressed. First, the drug response prediction models were trained using one pharmacogenomic database (CCLE), and we attempted to overcome this drawback by applying multiple GC datasets to the prediction model. Second, to explore the genomic features affecting the sensitivity to various drugs, numerous drug prediction models should be incorporated into a one-model approach for individual drugs. Third, genomic profiles may not have local patterns and genomic profile data may not be suitable for CNN model using stride. However, a recent study demonstrated that a CNN model using strides in unstructured genomic profile data improved the performance of a cancer type prediction model^57^. In this sense, CDRscan is a CNN model with strides. Fourth, some models have low correlation coefficients between the actual and predicted values, explainable gene features should be carefully interpreted.

Regarding modeling individual cancer drug response dataset and modeling the entire drug response dataset^14,58^, the two kinds of modeling serve different purposes, and the evaluation of the modeling results can vary depending on their intended usages. Modeling individual cancer drug response helps to identify gene features specific to a drug^58^. On the other hand, modeling the entire dataset helps to identify the most responsive drug in a patient among diverse drugs^14^. Therefore, modeling individual cancer drug response can be useful in identifying drug-specific informative genes^58^.

## Conclusion

Our research offers a useful guide for constructing a drug response prediction model using a one-model approach for individual drugs by integrating with XAI.

## Methods

### Data collection

Using CCLE^2^, we collected drug screening data as cell viability IC_50_, which is the half-maximal inhibitory concentrations, from cell viability assays in cancer cell lines (Fig. 1). For 24 drugs, IC_50_ data were available for 504 cancer cell lines in CCLE (version 24 Feb 2015). The screening concentrations for CCLE ranged between 0.0025 and 8.0 μM. In addition, we obtained mutation profile data from GDSC^3,25^. The mutation profile data consisted of 21,213 mutation sites in 1001 cell lines. The mRNA expression profiles of 18,988 genes in 1,037 cell lines were obtained from CCLE.

To generate combination datasets (Supplementary Table S1), we used cell line *e*xpression profiles (denoted as “E”), cell line *m*utation statuses (denoted as “M”), and drug response measurements as ln(IC_50_)s from *C*CLE (denoted as “C”)^2^.

The sources of the downloaded datasets are described in Supplementary Method S1.

### Setting 1: construction of a one-model approach for an individual drug, considering *e*xpression profiles (EC-11K) as input for prediction of ln(IC_50_)s

The EC-11K dataset for setting 1 consisted of the *e*xpression profiles of 504 *C*CLE cancer cell lines as input and ln(IC_50_) values of 24 drugs examined in cancer cell lines as output (Supplementary Table S1). Accordingly, 11,360 (~ *11K*) ln(IC_50_) measurements were available for cell line-drug treatment pairs from *C*CLE. In the dataset, the input vector had z-normalized expression elements for 18,988 genes (“EC-11K”, Supplementary Table S1). We then obtained 24 data matrices for 24 drugs from the EC-11K. Each data matrix was randomly divided into training and test sets at a ratio of 8 to 2 (Supplementary Fig. S1a and Supplementary Table S2).

### Setting 2: construction of one-model approach, considering *m*utation as input for predicting ln(IC_50_)s, from dataset MC-9K

MC-9K, the combined dataset, comprised mutational statuses for 504 cancer cell lines, amounting to 21,213 mutation positions (point mutations) as inputs and ln(IC_50_) values of 24 drugs as outputs (Supplementary Table S1). For each cancer cell line, the mutational statuses for 21,213 mutation sites were binarized to either 1 (presence) or 0 (absence). As a result, the number of features (mutations) in an the input vector was 21,213 for the dataset of 8727 (~ 9K) ln(IC_50_) measurements (“MC-9K”, Supplementary Table S1).

Likewise to setting 1, in the MC-9K dataset, 24 data matrices for 24 drugs were obtained, and each data matrix was randomly split into training and test sets at a ratio of 8 to 2 (Supplementary Fig. S1b and Supplementary Table S3).

### Construction of DL and ML models

We adopted CNN and ResNet architectures for regression-based DL models. For CNN architecture, we modified the architecture of the ‘CDRscan master’ model^15^ by eliminating CNN layers for drugs (Fig. 2a, Supplementary Fig. S21, and Supplementary Tables S20 and S21).

ResNet architecture (Fig. 2b, Supplementary Tables S22 and S23) was adopted from ResNetIC50, as previously reported^14,35^. The ResNet had 30 layers, including 9 skip connections (Fig. 2b and Supplementary Fig. S22).

For both the CNN and ResNet architectures, the loss function was the mean square error, while the rectified linear activation (ReLU) or the hyperbolic tangent function was used as the activation function. The CNN and ResNet parameters are indicated in Supplementary Tables S24 and S25.

We employed lasso, ridge, RF, SVR, and XGBoost for ML models. In all settings, the hyperparameter was set to alpha 0.001 for ridge and lasso, C 0.01 for SVR, and default options for RF and XGBoost (Supplementary Tables S24 and S25).

### Model performance comparisons

For model performance comparisons, we calculated R^2^ and RMSE values between the predicted and the observed ln(IC_50_) values in the test set. The formulas are as follows:

1 $$\mathrm{R}^{2} = 1-\frac{\sum_{\rm{i}=1}^{\mathrm{N}}{\left({\mathrm{y}}_{\rm{i}}-{\mathrm{f}}_{\rm{i}}\right)}^{2}}{\sum_{\rm{i}=1}^{\mathrm{N}}{\left({\mathrm{y}}_{\rm{i}}-\overline{\mathrm{y} }\right)}^{2}},$$

2 $$\mathrm{RMSE }=\sqrt{\frac{1}{\mathrm{N}}\sum_{\rm{i}=1}^{\mathrm{N}}{\left({\mathrm{y}}_{\rm{i}}-{\mathrm{f}}_{\rm{i}}\right)}^{2},}$$

where N is the number of cell lines in the test set; *y*_*i*_ is the *i*th observed ln(IC_50_); and *f*_*i*_ is the predicted ln(IC50) for the *i*th case. Thus, $\overline{\mathrm{y} }$ indicates the average of all *y* values. A scatter plot was adopted to visualize predicted and observed ln(IC_50_) values in the test set.

### Application of ridge for panobinostat from setting 1 to GC cell lines and patient datasets

Considering the performance comparisons, we confirmed that the ridge model for panobinostat in setting 1 exhibited superior performance to other models in all settings. Then, to confirm the applicability of this model, we obtained gene expression profiles of GC cell lines from CCLE (n = 18)^2^ and GDSC (n = 24)^3,25^.

As cell lines are recognized as potential proxies for drug development^2,10,59,60^, we assumed that patients’ drug response correlated with cell line-drug response.

For this purpose, we also obtained gene expression profiles of patients with GC from GSE118916 (n = 15)^26^ and The Cancer Genome Atlas of Stomach Adenocarcinoma (TCGA-STAD) from UCSC XENA (IlluminaHiSeq pancan normalized [n = 450])^27,61^. The gene expression profiles from the four GC datasets were also z-normalized; then, the predicted ln(IC_50_)s for panobinostat were obtained by inputting the z-normalized gene expression profiles into the ridge model for panobinostat. Using predicted ln(IC_50_)s values in the four GC datasets, we selected the four sensitive cases for panobinostat from the GC datasets, respectively (case A for CCLE GC, case B for GDSC GC, case C for GSE118916 GC, and case D for TCGA GC).

For CCLE and GDSC GC cell lines, we performed pearson’s correlation analysis between the predicted ln(IC_50_) values and the observed ln(IC_50_) values to investigate the applicability of the ridge model for panobinostat in setting 1 to GC cell lines and patients.

### Inspection of the major genomic features affecting drug response prediction using LIME analysis

To explore explainable genomic features of the ridge model for panobinostat in setting 1, we adopted LIME for an XAI method^62^ to the ridge model for panobinostat, obtaining important genes in the model using the four selected GC cases (cases A through D). For LIME, the python lime package^62^ was used with default parameter settings, with 18,988 explainable features. Then, based on the LIME analysis, the top three explainable biological (or genomic) features affecting drug response and the top three features affecting drug response were yielded in each case (cases A through D).

Also, we compared the gene features selected by application of XAI to the panobinostat ridge model (setting 1) in the four cases (cases A through D) with those selected in the panobinostat ridge model (setting 1). The top 100 gene features by XAI in each case were merged to obtain 374 non-redundant gene features. Subsequently, the non-redundant gene features selected by XAI were compared with the 100 gene features selected in the panobinostat ridge model.

## Supplementary Information

Supplementary Information.

## Data Availability

The data underlying this article are available on GitHub at https://github.com/labnams/IC50_individual_drug.
