# Supplementary material for: A performance evaluation of drug response prediction models for individual drugs
Source: Sci Rep. 2023 Jul 24;13:11911. doi: 10.1038/s41598-023-39179-2 (PMC10366128; doi:10.1038/s41598-023-39179-2)
Supplement: Supplementary file 1 — Supplementary Information. [file 41598_2023_39179_MOESM1_ESM.pdf]

A performance evaluation of drug response prediction models for individual drugs

Park *et al*.

Supplementary Information

Supplementary Method S1.

Supplementary Tables S1 through S25

Supplementary Figures S1 through S22

Supplementary References

Supplementary Method S1. Raw data description

The mutation profile was downloaded from GDSC (Release-7.0; last modified on Mar 20, 2018) [1], and the profile is in ENSEMBL annotation version 56 (ftp://ftp.sanger.ac.uk/pub4/cancerrxgene/releases/release-7.0/WES_variants.xlsx). The expression profile was obtained from Cancer Cell Line Encyclopedia (CCLE) [2], uploaded on Oct 17, 2012 (https://data.broadinstitute.org/ccle_legacy_data/mRNA_expression/CCLE_Expression_Entrez_2012-09-29.gct). Drug response measurement (IC_{50}) data from CCLE (Released on Feb 24, 2015) were downloaded via https://data.broadinstitute.org/ccle_legacy_data/pharmacological_profiling/CCLE_NP24.2009_Drug_data_2015.02.24.csv.

Gene expression data of gastric cancer cell lines from GDSC was downloaded via the GDSC1000 resource (https://www.cancerrxgene.org/gdsc1000/GDSC1000_WebResources//Data/preprocessed/Cell_line_RMA_proc_basalExp.txt.zip).

Supplementary Table S1. Description of datasets. We set various combinations of dataset size and genomic information and nominally assigned each combination to a dataset name (first column).

Dataset names	Input of genomics information	Genomics vector length	Output (observed cell viability)	Dataset size
EC-11K	Expression (CCLE)	18,988 (genes)	ln(IC50)s from CCLE	11,360
MC-9K	Mutation (GDSC)	21,213 (mutation positions)	ln(IC50)s from CCLE	8,727

Supplementary Table S2. The number of samples in the training and test sets for the models of 24 individual drugs in setting 1.

Drug	Samples for training	Samples for test
AEW541	392	98
Nilotinib	326	81
17-AAG	392	98
PHA-665752	392	98
Lapatinib	393	98
Nutlin-3	393	98
AZD0530	393	98
PF2341066	393	98
L-685458	383	95
ZD-6474	387	96
Panobinostat	390	97
Sorafenib	392	98
Irinotecan	245	61
Topotecan	393	98
LBW242	392	98
PD-0325901	393	98
PD-0332991	337	84
Paclitaxel	392	98
PLX4720	387	96
RAF265	358	89
TAE684	393	98
TKI258	392	98
Erlotinib	392	98
AZD6244	392	98

Supplementary Table S3. The number of samples in the training and test sets for the models of 24 individual drugs in setting 2.

Drug	Samples for training	Samples for test
AEW541	303	75
Nilotinib	248	61
17-AAG	302	75
PHA-665752	302	75
Lapatinib	303	75
Nutlin-3	303	75
AZD0530	303	75
PF2341066	303	75
L-685458	292	73
ZD-6474	298	74
Panobinostat	300	75
Sorafenib	302	75
Irinotecan	183	45
Topotecan	303	75
LBW242	302	75
PD-0325901	303	75
PD-0332991	258	64
Paclitaxel	302	75
PLX4720	298	74
RAF265	274	68
TAE684	303	75
TKI258	303	75
Erlotinib	303	75
AZD6244	302	75

Supplementary Table S4. Performance of the models for 24 individual drugs in setting 1, using Lasso.

Drug	RMSE	R^2
PD-0325901	1.716	0.345
Panobinostat	0.729	0.275
AZD6244	1.276	0.216
Topotecan	1.355	0.200
PLX4720	0.609	0.176
PF2341066	0.616	0.137
17-AAG	1.266	0.130
Irinotecan	1.180	0.106
RAF265	1.206	0.099
Paclitaxel	2.196	0.071
L-685458	0.724	0.063
Lapatinib	0.803	0.059
ZD-6474	0.799	0.002
PD-0332991	0.617	0.000
Nutlin-3	0.811	-0.039
TAE684	1.400	-0.046
PHA-665752	0.880	-0.101
AEW541	0.920	-0.333
TKI258	0.806	-0.341
Sorafenib	0.850	-0.448
AZD0530	0.866	-0.495
Nilotinib	0.698	-0.620
Erlotinib	0.719	-0.688
LBW242	0.645	-4.864

R^2, R-squared value; RMSE, root mean squared error.

Supplementary Table S5. Performance of the models for 24 individual drugs in setting 1, using ridge.

Drug	RMSE	R^2
PD-0325901	1.632	0.408
Panobinostat	0.623	0.470
AZD6244	1.137	0.378
Topotecan	1.298	0.266
PLX4720	0.577	0.260
PF2341066	0.604	0.169
17-AAG	1.249	0.153
Irinotecan	1.201	0.075
RAF265	1.067	0.294
Paclitaxel	2.282	-0.004
L-685458	0.672	0.194
Lapatinib	0.652	0.379
ZD-6474	0.777	0.056
PD-0332991	0.529	0.265
Nutlin-3	0.804	-0.022
TAE684	1.223	0.202
PHA-665752	0.851	-0.027
AEW541	0.827	-0.078
TKI258	0.700	-0.010
Sorafenib	0.786	-0.238
AZD0530	0.798	-0.271
Nilotinib	0.682	-0.545
Erlotinib	0.624	-0.270
LBW242	0.472	-2.144

R^2, R-squared value; RMSE, root mean squared error.

Supplementary Table S6. Performance of the models for 24 individual drugs in setting 1, using SVR.

Drug	RMSE	R^2
PD-0325901	2.564	-0.462
Panobinostat	0.837	0.043
AZD6244	1.559	-0.170
Topotecan	1.513	0.002
PLX4720	0.676	-0.015
PF2341066	0.684	-0.066
17-AAG	1.374	-0.025
Irinotecan	1.245	0.006
RAF265	1.337	-0.107
Paclitaxel	2.356	-0.070
L-685458	0.762	-0.038
Lapatinib	0.883	-0.138
ZD-6474	0.872	-0.187
PD-0332991	0.623	-0.018
Nutlin-3	0.796	0.000
TAE684	1.386	-0.026
PHA-665752	0.845	-0.015
AEW541	0.861	-0.167
TKI258	0.783	-0.265
Sorafenib	0.711	-0.014
AZD0530	0.737	-0.082
Nilotinib	0.553	-0.015
Erlotinib	0.562	-0.032
LBW242	0.274	-0.057

R^2, R-squared value; RMSE, root mean squared error.

Supplementary Table S7. Performance of the models for 24 individual drugs in setting 1, using random forest.

Drug	RMSE	R^2
PD-0325901	1.664	0.384
Panobinostat	0.644	0.434
AZD6244	1.176	0.334
Topotecan	1.226	0.345
PLX4720	0.620	0.144
PF2341066	0.572	0.254
17-AAG	1.159	0.270
Irinotecan	0.995	0.365
RAF265	1.203	0.104
Paclitaxel	2.140	0.118
L-685458	0.678	0.178
Lapatinib	0.714	0.257
ZD-6474	0.748	0.125
PD-0332991	0.502	0.338
Nutlin-3	0.798	-0.006
TAE684	1.245	0.173
PHA-665752	0.836	0.008
AEW541	0.808	-0.028
TKI258	0.621	0.206
Sorafenib	0.720	-0.041
AZD0530	0.706	0.005
Nilotinib	0.601	-0.199
Erlotinib	0.520	0.115
LBW242	0.451	-1.870

R^2, R-squared value; RMSE, root mean squared error.

Supplementary Table S8. Performance of the models for 24 individual drugs in setting 1, using XGBoost.

Drug	RMSE	R^2
PD-0325901	1.894	0.202
Panobinostat	0.710	0.311
AZD6244	1.279	0.213
Topotecan	1.378	0.172
PLX4720	0.697	-0.081
PF2341066	0.666	-0.009
17-AAG	1.340	0.024
Irinotecan	1.080	0.251
RAF265	1.249	0.032
Paclitaxel	2.131	0.125
L-685458	0.663	0.214
Lapatinib	0.705	0.274
ZD-6474	0.767	0.081
PD-0332991	0.523	0.283
Nutlin-3	0.802	-0.017
TAE684	1.292	0.108
PHA-665752	0.821	0.043
AEW541	0.840	-0.112
TKI258	0.722	-0.076
Sorafenib	0.718	-0.034
AZD0530	0.736	-0.079
Nilotinib	0.741	-0.822
Erlotinib	0.617	-0.243
LBW242	0.804	-8.113

R^2, R-squared value; RMSE, root mean squared error.

Supplementary Table S9. Performance of the models for 24 individual drugs in setting 1, using ElasticNet.

Drug	RMSE	R^2
PD-0325901	1.655	0.391
Panobinostat	0.715	0.302
Irinotecan	1.153	0.147
Topotecan	1.299	0.265
AZD6244	1.209	0.296
PLX4720	0.573	0.269
PD-0332991	0.626	-0.030
TKI258	0.781	-0.258
Lapatinib	0.776	0.121
TAE684	1.393	-0.036
AZD0530	0.830	-0.375
L-685458	0.726	0.057
PF2341066	0.612	0.147
17-AAG	1.273	0.120
RAF265	1.185	0.130
AEW541	0.907	-0.296
Nilotinib	0.700	-0.630
Nutlin-3	0.811	-0.038
ZD-6474	0.818	-0.046
Erlotinib	0.696	-0.582
PHA-665752	0.881	-0.103
Sorafenib	0.840	-0.416
Paclitaxel	2.238	0.035
LBW242	0.628	-4.556

R^2, R-squared value; RMSE, root mean squared error.

Supplementary Table S10. Performance of the models for 24 individual drugs in setting 1, using ResNet.

Drug	RMSE	R^2
PD-0325901	2.214	-0.091
Panobinostat	1.121	-0.716
AZD6244	1.502	-0.086
Topotecan	1.560	-0.060
PLX4720	0.750	-0.251
PF2341066	0.667	-0.011
17-AAG	1.367	-0.015
Irinotecan	1.344	-0.159
RAF265	1.307	-0.059
Paclitaxel	3.563	-1.446
L-685458	0.785	-0.102
Lapatinib	0.952	-0.322
ZD-6474	0.819	-0.049
PD-0332991	0.692	-0.257
Nutlin-3	0.797	-0.003
TAE684	1.424	-0.082
PHA-665752	0.897	-0.142
AEW541	0.930	-0.363
TKI258	0.824	-0.398
Sorafenib	0.798	-0.277
AZD0530	0.781	-0.215
Nilotinib	0.578	-0.109
Erlotinib	0.643	-0.352
LBW242	0.517	-2.763

R^2, R-squared value; RMSE, root mean squared error.

Supplementary Table S11. Performance of the models for 24 individual drugs in setting 1, using CNN.

Drug	RMSE	R^2
PD-0325901	1.734	0.331
Panobinostat	0.709	0.314
AZD6244	1.288	0.201
Topotecan	1.337	0.222
PLX4720	0.609	0.176
PF2341066	0.640	0.067
17-AAG	1.312	0.065
Irinotecan	1.092	0.235
RAF265	1.236	0.054
Paclitaxel	2.404	-0.114
L-685458	0.715	0.086
Lapatinib	0.776	0.122
ZD-6474	0.811	-0.027
PD-0332991	0.561	0.175
Nutlin-3	0.797	-0.003
TAE684	1.292	0.109
PHA-665752	0.855	-0.038
AEW541	0.784	0.031
TKI258	0.645	0.143
Sorafenib	0.723	-0.047
AZD0530	0.676	0.088
Nilotinib	0.546	0.011
Erlotinib	0.563	-0.034
LBW242	0.284	-0.133

R^2, R-squared value; RMSE, root mean squared error.

Supplementary Table S12. Performance of the models for 24 individual drugs in setting 2, using Lasso.

Drug	RMSE	R^2
Nilotinib	0.769	0.393
PHA-665752	0.365	-0.093
AZD6244	1.595	-0.008
PD-0325901	2.359	-0.137
Panobinostat	0.833	-0.015
Nutlin-3	0.852	-0.023
PD-0332991	0.543	-0.065
Paclitaxel	2.110	-0.129
LBW242	0.875	-0.111
ZD-6474	0.920	-0.160
Irinotecan	1.484	-0.252
TAE684	1.237	-0.181
RAF265	1.220	-0.129
AEW541	0.956	-0.246
L-685458	0.810	-0.154
AZD0530	0.969	-0.217
Topotecan	1.714	-0.267
17-AAG	1.972	-0.328
Erlotinib	0.749	-0.255
TKI258	0.653	-0.261
PF2341066	0.798	-0.385
Lapatinib	0.696	-0.195
Sorafenib	0.459	-0.319
PLX4720	0.521	-1.417

R^2, R-squared value; RMSE, root mean squared error.

Supplementary Table S13. Performance of the models for 24 individual drugs in setting 2, using ridge.

Drug	RMSE	R^2
Nilotinib	0.969	0.038
PHA-665752	0.346	0.015
AZD6244	1.500	0.107
PD-0325901	2.291	-0.073
Panobinostat	0.894	-0.167
Nutlin-3	0.853	-0.025
PD-0332991	1.457	-6.660
Paclitaxel	2.321	-0.366
LBW242	0.857	-0.068
ZD-6474	0.855	-0.002
Irinotecan	1.858	-0.963
TAE684	1.133	0.009
RAF265	1.960	-1.914
AEW541	0.844	0.029
L-685458	1.158	-1.358
AZD0530	0.882	-0.009
Topotecan	2.010	-0.743
17-AAG	1.801	-0.108
Erlotinib	0.957	-1.049
TKI258	0.811	-0.943
PF2341066	0.811	-0.429
Lapatinib	1.154	-2.289
Sorafenib	0.426	-0.137
PLX4720	0.779	-4.397

R^2, R-squared value; RMSE, root mean squared error.

Supplementary Table S14. Performance of the models for 24 individual drugs in setting 2, using random forest.

Drug	RMSE	R^2
Nilotinib	0.808	0.330
PHA-665752	0.354	-0.028
AZD6244	1.490	0.119
PD-0325901	2.188	0.021
Panobinostat	0.836	-0.022
Nutlin-3	0.853	-0.024
PD-0332991	1.097	-3.343
Paclitaxel	2.342	-0.391
LBW242	0.853	-0.056
ZD-6474	0.879	-0.058
Irinotecan	1.781	-0.803
TAE684	1.183	-0.081
RAF265	1.360	-0.404
AEW541	0.893	-0.087
L-685458	1.090	-1.089
AZD0530	0.907	-0.068
Topotecan	2.061	-0.832
17-AAG	1.822	-0.134
Erlotinib	0.788	-0.390
TKI258	0.804	-0.910
PF2341066	0.690	-0.033
Lapatinib	1.027	-1.603
Sorafenib	0.422	-0.115
PLX4720	0.748	-3.972

R^2, R-squared value; RMSE, root mean squared error.

Supplementary Table S15. Performance of the models for 24 individual drugs in setting 2, using SVR.

Drug	RMSE	R^2
Nilotinib	1.019	-0.065
PHA-665752	0.349	0.000
AZD6244	1.742	-0.203
PD-0325901	2.697	-0.486
Panobinostat	0.828	-0.002
Nutlin-3	0.843	-0.002
PD-0332991	0.531	-0.017
Paclitaxel	2.002	-0.017
LBW242	0.835	-0.013
ZD-6474	0.924	-0.169
Irinotecan	1.330	-0.005
TAE684	1.193	-0.099
RAF265	1.222	-0.134
AEW541	1.003	-0.371
L-685458	0.762	-0.021
AZD0530	0.951	-0.173
Topotecan	1.534	-0.014
17-AAG	1.723	-0.014
Erlotinib	0.681	-0.039
TKI258	0.672	-0.335
PF2341066	0.750	-0.224
Lapatinib	0.663	-0.086
Sorafenib	0.406	-0.035
PLX4720	0.336	-0.005

R^2, R-squared value; RMSE, root mean squared error.

Supplementary Table S16. Performance of the models for 24 individual drugs in setting 2, using XGBoost.

Drug	RMSE	R^2
Nilotinib	0.772	0.389
PHA-665752	0.323	0.146
AZD6244	1.475	0.138
PD-0325901	2.159	0.047
Panobinostat	0.822	0.012
Nutlin-3	0.856	-0.032
PD-0332991	0.538	-0.042
Paclitaxel	2.124	-0.145
LBW242	0.891	-0.154
ZD-6474	0.926	-0.175
Irinotecan	1.446	-0.188
TAE684	1.245	-0.197
RAF265	1.256	-0.198
AEW541	0.941	-0.207
L-685458	0.830	-0.212
AZD0530	0.982	-0.251
Topotecan	1.706	-0.255
17-AAG	1.934	-0.277
Erlotinib	0.783	-0.372
TKI258	0.691	-0.413
PF2341066	0.808	-0.418
Lapatinib	0.779	-0.500
Sorafenib	0.503	-0.588
PLX4720	0.562	-1.809

R^2, R-squared value; RMSE, root mean squared error.

Supplementary Table S17. Performance of the models for 24 individual drugs in setting 2, using ElasticNet.

Drug	RMSE	R^2
Nilotinib	0.888	0.192
PHA-665752	0.330	0.106
AZD6244	1.766	-0.237
PD-0325901	2.437	-0.214
Panobinostat	0.817	0.025
Nutlin-3	0.854	-0.027
PD-0332991	0.911	-1.994
Paclitaxel	2.240	-0.272
LBW242	0.958	-0.334
ZD-6474	0.947	-0.228
Irinotecan	1.736	-0.714
TAE684	1.210	-0.131
RAF265	1.542	-0.803
AEW541	0.912	-0.135
L-685458	0.967	-0.644
AZD0530	0.955	-0.182
Topotecan	2.043	-0.800
17-AAG	1.898	-0.230
Erlotinib	0.708	-0.122
TKI258	0.734	-0.595
PF2341066	0.674	0.013
Lapatinib	1.003	-1.483
Sorafenib	0.468	-0.376
PLX4720	0.619	-2.403

R^2, R-squared value; RMSE, root mean squared error.

Supplementary Table S18. Performance of the models for 24 individual drugs in setting 2, using ResNet.

Drug	RMSE	R^2
Nilotinib	1.005	-0.035
PHA-665752	0.389	-0.244
AZD6244	1.592	-0.005
PD-0325901	2.239	-0.025
Panobinostat	1.210	-1.141
Nutlin-3	0.962	-0.303
PD-0332991	0.630	-0.431
Paclitaxel	2.952	-1.210
LBW242	0.847	-0.041
ZD-6474	0.870	-0.036
Irinotecan	1.446	-0.188
TAE684	1.244	-0.196
RAF265	1.185	-0.066
AEW541	0.925	-0.167
L-685458	0.857	-0.290
AZD0530	0.919	-0.096
Topotecan	1.538	-0.020
17-AAG	1.880	-0.207
Erlotinib	0.703	-0.106
TKI258	0.608	-0.095
PF2341066	0.944	-0.939
Lapatinib	0.669	-0.104
Sorafenib	0.632	-1.509
PLX4720	0.847	-5.377

R^2, R-squared value; RMSE, root mean squared error.

Supplementary Table S19. Performance of the models for 24 individual drugs in setting 2, using CNN.

Drug	RMSE	R^2
Nilotinib	0.994	-0.014
PHA-665752	0.360	-0.063
AZD6244	1.627	-0.049
PD-0325901	2.224	-0.011
Panobinostat	0.837	-0.024
Nutlin-3	0.848	-0.012
PD-0332991	0.536	-0.036
Paclitaxel	1.982	0.004
LBW242	0.837	-0.018
ZD-6474	0.856	-0.004
Irinotecan	1.326	0.001
TAE684	1.142	-0.008
RAF265	1.188	-0.072
AEW541	0.852	0.009
L-685458	0.765	-0.028
AZD0530	0.891	-0.030
Topotecan	1.575	-0.070
17-AAG	1.713	-0.002
Erlotinib	0.661	0.023
TKI258	1.686	-7.405
PF2341066	0.679	-0.002
Lapatinib	0.634	0.008
Sorafenib	0.404	-0.022
PLX4720	0.390	-0.353

R^2, R-squared value; RMSE, root mean squared error.

Supplementary Table S20. Model architecture and respective parameters in the CNN model (setting 1).

Layer (type)	Output Shape	Param #
**cell_input (InputLayer)**	(None, 18988, 1)	0
**conv1d_1 (Conv1D)**	(None, 3658, 50)	35050
**max_pooling1d_1 (MaxPooling1D)**	(None, 731, 50)	0
**conv1d_2 (Conv1D)**	(None, 364, 30)	7530
**max_pooling1d_2 (MaxPooling1D)**	(None, 36, 30)	0
**flatten_1 (Flatten)**	(None, 1080)	0
**dense_1 (Dense)**	(None, 100)	108100
**dropout_1 (Dropout)**	(None, 100)	0
**dense_2 (Dense)**	(None, 300)	30300
**dropout_2 (Dropout)**	(None, 300)	0
**reshape_1 (Reshape)**	(None, 300, 1)	0
**conv1d_3 (Conv1D)**	(None, 151, 30)	4530
**max_pooling1d_3 (MaxPooling1D)**	(None, 75, 30)	0
**conv1d_4 (Conv1D)**	(None, 71, 10)	1510
**max_pooling1d_4 (MaxPooling1D)**	(None, 23, 10)	0
**conv1d_5 (Conv1D)**	(None, 19, 5)	255
**max_pooling1d_5 (MaxPooling1D)**	(None, 6, 5)	0
**dropout_3 (Dropout)**	(None, 6, 5)	0
**flatten_2 (Flatten)**	(None, 30)	0
**dropout_4 (Dropout)**	(None, 30)	0
**pred_lnIC50 (Dense)**	(None, 1)	31

Total params: 187,306
Trainable params: 187,306
Non-trainable params: 0

Supplementary Table S21. Model architecture and respective parameters in the CNN model (setting 2).

Layer (type)	Output Shape	Param #
**cell_input (InputLayer)**	(None, 21213, 1)	0
**conv1d_1 (Conv1D)**	(None, 4103, 50)	35050
**max_pooling1d_1 (MaxPooling1D)**	(None, 820, 50)	0
**conv1d_2 (Conv1D)**	(None, 408, 30)	7530
**max_pooling1d_2 (MaxPooling1D)**	(None, 40, 30)	0
**flatten_1 (Flatten)**	(None, 1200)	0
**dense_1 (Dense)**	(None, 100)	120100
**dropout_1 (Dropout)**	(None, 100)	0
**dense_2 (Dense)**	(None, 300)	30300
**dropout_2 (Dropout)**	(None, 300)	0
**reshape_1 (Reshape)**	(None, 300, 1)	0
**conv1d_3 (Conv1D)**	(None, 151, 30)	4530
**max_pooling1d_3 (MaxPooling1D)**	(None, 75, 30)	0
**conv1d_4 (Conv1D)**	(None, 71, 10)	1510
**max_pooling1d_4 (MaxPooling1D)**	(None, 23, 10)	0
**conv1d_5 (Conv1D)**	(None, 19, 5)	255
**max_pooling1d_5 (MaxPooling1D)**	(None, 6, 5)	0
**dropout_3 (Dropout)**	(None, 6, 5)	0

**flatten_2 (Flatten)**	(None, 30)	0
**dropout_4 (Dropout)**	(None, 30)	0
**pred_lnIC50 (Dense)**	(None, 1)	31
**Total params: 199,306**<br>**Trainable params: 199,306**<br>**Non-trainable params: 0**		

Supplementary Table S22. Model architecture and respective parameters in the ResNet model (setting 1).

**Layer (type)**	Output Shape	Param #	Connected to
**inputs (InputLayer)**	(None, 18988, 1)	0	
**conv1d_1 (Conv1D)**	(None, 9494, 16)	64	inputs[0][0]
**max_pooling1d_1 (MaxPooling1D)**	(None, 1898, 16)	0	conv1d_1[0][0]
**batch_normalization_1 (BatchNormalization)**	(None, 1898, 16)	64	max_pooling1d_1[0][0]
**activation_1 (Activation)**	(None, 1898, 16)	0	batch_normalization_1[0][0]
**conv1d_2 (Conv1D)**	(None, 1898, 16)	784	activation_1[0][0]
**batch_normalization_2 (BatchNormalization)**	(None, 1898, 16)	64	conv1d_2[0][0]
**activation_2 (Activation)**	(None, 1898, 16)	0	batch_normalization_2[0][0]
**conv1d_3 (Conv1D)**	(None, 1898, 16)	784	activation_2[0][0]
**batch_normalization_3 (BatchNormalization)**	(None, 1898, 16)	64	conv1d_3[0][0]
**activation_3 (Activation)**	(None, 1898, 16)	0	batch_normalization_3[0][0]
**conv1d_4 (Conv1D)**	(None, 1898, 16)	784	activation_3[0][0]
**batch_normalization_4 (BatchNormalization)**	(None, 1898, 16)	64	conv1d_4[0][0]
**add_1 (Add)**	(None, 1898, 16)	0	batch_normalization_4[0][0]<br>batch_normalization_2[0][0]
**activation_4 (Activation)**	(None, 1898, 16)	0	add_1[0][0]
**conv1d_5 (Conv1D)**	(None, 1898, 16)	784	activation_4[0][0]
**batch_normalization_5 (BatchNormalization)**	(None, 1898, 16)	64	conv1d_5[0][0]

**activation_5 (Activation)**	(None, 1898, 16)	0	batch_normalization_5[0][0]
**conv1d_6 (Conv1D)**	(None, 1898, 16)	784	activation_5[0][0]
**batch_normalization_6 (BatchNormalization)**	(None, 1898, 16)	64	conv1d_6[0][0]
**activation_6 (Activation)**	(None, 1898, 16)	0	batch_normalization_6[0][0]
**conv1d_7 (Conv1D)**	(None, 1898, 16)	784	activation_6[0][0]
**batch_normalization_7 (BatchNormalization)**	(None, 1898, 16)	64	conv1d_7[0][0]
**activation_7 (Activation)**	(None, 1898, 16)	0	batch_normalization_7[0][0]
**conv1d_8 (Conv1D)**	(None, 1898, 16)	784	activation_7[0][0]
**batch_normalization_8 (BatchNormalization)**	(None, 1898, 16)	64	conv1d_8[0][0]
**add_2 (Add)**	(None, 1898, 16)	0	batch_normalization_8[0][0]<br>batch_normalization_5[0][0]
**activation_8 (Activation)**	(None, 1898, 16)	0	add_2[0][0]
**conv1d_9 (Conv1D)**	(None, 949, 32)	1568	activation_8[0][0]
**batch_normalization_9 (BatchNormalization)**	(None, 949, 32)	128	conv1d_9[0][0]
**activation_9 (Activation)**	(None, 949, 32)	0	batch_normalization_9[0][0]
**conv1d_10 (Conv1D)**	(None, 949, 32)	3104	activation_9[0][0]
**conv1d_11 (Conv1D)**	(None, 949, 32)	3104	conv1d_10[0][0]
**batch_normalization_10 (BatchNormalization)**	(None, 949, 32)	128	conv1d_11[0][0]
**add_3 (Add)**	(None, 949, 32)	0	batch_normalization_10[0][0]<br>batch_normalization_9[0][0]
**activation_10 (Activation)**	(None, 949, 32)	0	add_3[0][0]

**conv1d_12 (Conv1D)**	(None, 949, 32)	3104	activation_10[0][0]
**batch_normalization_11 (BatchNormalization)**	(None, 949, 32)	128	conv1d_12[0][0]
**activation_11 (Activation)**	(None, 949, 32)	0	batch_normalization_11[0][0]
**conv1d_13 (Conv1D)**	(None, 949, 32)	3104	activation_11[0][0]
**batch_normalization_12 (BatchNormalization)**	(None, 949, 32)	128	conv1d_13[0][0]
**add_4 (Add)**	(None, 949, 32)	0	batch_normalization_12[0][0]<br>batch_normalization_11[0][0]
**activation_12 (Activation)**	(None, 949, 32)	0	add_4[0][0]
**conv1d_14 (Conv1D)**	(None, 949, 32)	3104	activation_12[0][0]
**batch_normalization_13 (BatchNormalization)**	(None, 949, 32)	128	conv1d_14[0][0]
**activation_13 (Activation)**	(None, 949, 32)	0	batch_normalization_13[0][0]
**conv1d_15 (Conv1D)**	(None, 949, 32)	3104	activation_13[0][0]
**batch_normalization_14 (BatchNormalization)**	(None, 949, 32)	128	conv1d_15[0][0]
**add_5 (Add)**	(None, 949, 32)	0	batch_normalization_14[0][0]<br>batch_normalization_13[0][0]
**activation_14 (Activation)**	(None, 949, 32)	0	add_5[0][0]
**conv1d_16 (Conv1D)**	(None, 475, 64)	6208	activation_14[0][0]
**batch_normalization_15 (BatchNormalization)**	(None, 475, 64)	256	conv1d_16[0][0]
**activation_15 (Activation)**	(None, 475, 64)	0	batch_normalization_15[0][0]
**conv1d_17 (Conv1D)**	(None, 475, 64)	12352	activation_15[0][0]
**conv1d_18 (Conv1D)**	(None, 475, 64)	12352	conv1d_17[0][0]

**batch_normalization_16 (BatchNormalization)**	(None, 475, 64)	256	conv1d_18[0][0]
**add_6 (Add)**	(None, 475, 64)	0	batch_normalization_16[0][0]<br>batch_normalization_15[0][0]
**activation_16 (Activation)**	(None, 475, 64)	0	add_6[0][0]
**conv1d_19 (Conv1D)**	(None, 475, 64)	12352	activation_16[0][0]
**batch_normalization_17 (BatchNormalization)**	(None, 475, 64)	256	conv1d_19[0][0]
**activation_17 (Activation)**	(None, 475, 64)	0	batch_normalization_17[0][0]
**conv1d_20 (Conv1D)**	(None, 475, 64)	12352	activation_17[0][0]
**batch_normalization_18 (BatchNormalization)**	(None, 475, 64)	256	conv1d_20[0][0]
**add_7 (Add)**	(None, 475, 64)	0	batch_normalization_18[0][0]<br>batch_normalization_17[0][0]
**activation_18 (Activation)**	(None, 475, 64)	0	add_7[0][0]
**conv1d_21 (Conv1D)**	(None, 475, 64)	12352	activation_18[0][0]
**batch_normalization_19 (BatchNormalization)**	(None, 475, 64)	256	conv1d_21[0][0]
**activation_19 (Activation)**	(None, 475, 64)	0	batch_normalization_19[0][0]
**conv1d_22 (Conv1D)**	(None, 475, 64)	12352	activation_19[0][0]
**batch_normalization_20 (BatchNormalization)**	(None, 475, 64)	256	conv1d_22[0][0]
**add_8 (Add)**	(None, 475, 64)	0	batch_normalization_20[0][0]<br>batch_normalization_19[0][0]
**activation_20 (Activation)**	(None, 475, 64)	0	add_8[0][0]
**flatten_1 (Flatten)**	(None, 30400)	0	activation_20[0][0]
**dense1 (Dense)**	(None, 2048)	62261248	flatten_1[0][0]

**batch_normalization_21 (BatchNormalization)**	(None, 2048)	8192	dense1[0][0]
**dropout1 (Dropout)**	(None, 2048)	0	batch_normalization_21[0][0]
**activation_21 (Activation)**	(None, 2048)	0	dropout1[0][0]
**dense5 (Dense)**	(None, 1024)	2098176	activation_21[0][0]
**batch_normalization_22 (BatchNormalization)**	(None, 1024)	4096	dense5[0][0]
**dropout5 (Dropout)**	(None, 1024)	0	batch_normalization_22[0][0]
**activation_22 (Activation)**	(None, 1024)	0	dropout5[0][0]
**dense6 (Dense)**	(None, 512)	524800	activation_22[0][0]
**batch_normalization_23 (BatchNormalization)**	(None, 512)	2048	dense6[0][0]
**dropout6 (Dropout)**	(None, 512)	0	batch_normalization_23[0][0]
**activation_23 (Activation)**	(None, 512)	0	dropout6[0][0]
**dense7 (Dense)**	(None, 1024)	525312	activation_23[0][0]
**batch_normalization_24 (BatchNormalization)**	(None, 1024)	4096	dense7[0][0]
**dropout7 (Dropout)**	(None, 1024)	0	batch_normalization_24[0][0]
**add_9 (Add)**	(None, 1024)	0	dropout7[0][0]<br>batch_normalization_22[0][0]
**activation_24 (Activation)**	(None, 1024)	0	add_9[0][0]
**dense8 (Dense)**	(None, 512)	524800	activation_24[0][0]
**batch_normalization_25 (BatchNormalization)**	(None, 512)	2048	dense8[0][0]
**dropout8 (Dropout)**	(None, 512)	0	batch_normalization_25[0][0]
**activation_25 (Activation)**	(None, 512)	0	dropout8[0][0]
**dense9 (Dense)**	(None, 256)	131328	activation_25[0][0]
**batch_normalization_26 (BatchNormalization)**	(None, 256)	1024	dense9[0][0]

**dropout9 (Dropout)**	(None, 256)	0	batch_normalization_26[0][0]
**activation_26 (Activation)**	(None, 256)	0	dropout9[0][0]
**dense10 (Dense)**	(None, 128)	32896	activation_26[0][0]
**batch_normalization_27 (BatchNormalization)**	(None, 128)	512	dense10[0][0]
**dropout10 (Dropout)**	(None, 128)	0	batch_normalization_27[0][0]
**activation_27 (Activation)**	(None, 128)	0	dropout10[0][0]
**predictions (Dense)**	(None, 1)	129	activation_27[0][0]
**Total params: 66,229,585 Trainable params: 66,217,169 Non-trainable params: 12,416**			

Supplementary Table S23. Model architecture and respective parameter in the ResNet model (setting 2).

Layer (type)	Output Shape	Param #	Connected to
**inputs (InputLayer)**	(None, 21213, 1)	0	
**conv1d_1 (Conv1D)**	(None, 10607, 16)	64	inputs[0][0]
**max_pooling1d_1 (MaxPooling1D)**	(None, 2121, 16)	0	conv1d_1[0][0]
**batch_normalization_1 (Batch_normalization)**	(None, 2121, 16)	64	max_pooling1d_1[0][0]
**activation_1 (Activation)**	(None, 2121, 16)	0	batch_normalization_1[0][0]
**conv1d_2 (Conv1D)**	(None, 2121, 16)	784	activation_1[0][0]
**batch_normalization_2 (Batch_normalization)**	(None, 2121, 16)	64	conv1d_2[0][0]
**activation_2 (Activation)**	(None, 2121, 16)	0	batch_normalization_2[0][0]
**conv1d_3 (Conv1D)**	(None, 2121, 16)	784	activation_2[0][0]
**batch_normalization_3 (Batch_normalization)**	(None, 2121, 16)	64	conv1d_3[0][0]
**activation_3 (Activation)**	(None, 2121, 16)	0	batch_normalization_3[0][0]
**conv1d_4 (Conv1D)**	(None, 2121, 16)	784	activation_3[0][0]
**batch_normalization_4 (Batch_normalization)**	(None, 2121, 16)	64	conv1d_4[0][0]
**add_1 (Add)**	(None, 2121, 16)	0	batch_normalization_4[0][0]<br>batch_normalization_2[0][0]
**activation_4 (Activation)**	(None, 2121, 16)	0	add_1[0][0]
**conv1d_5 (Conv1D)**	(None, 2121, 16)	784	activation_4[0][0]

**batch_normalization_5 (Batch_normalization)**	(None, 2121, 16)	64	conv1d_5[0][0]
**activation_5 (Activation)**	(None, 2121, 16)	0	batch_normalization_5[0][0]
**conv1d_6 (Conv1D)**	(None, 2121, 16)	784	activation_5[0][0]
**batch_normalization_6 (Batch_normalization)**	(None, 2121, 16)	64	conv1d_6[0][0]
**activation_6 (Activation)**	(None, 2121, 16)	0	batch_normalization_6[0][0]
**conv1d_7 (Conv1D)**	(None, 2121, 16)	784	activation_6[0][0]
**batch_normalization_7 (Batch_normalization)**	(None, 2121, 16)	64	conv1d_7[0][0]
**activation_7 (Activation)**	(None, 2121, 16)	0	batch_normalization_7[0][0]
**conv1d_8 (Conv1D)**	(None, 2121, 16)	784	activation_7[0][0]
**batch_normalization_8 (Batch_normalization)**	(None, 2121, 16)	64	conv1d_8[0][0]
**add_2 (Add)**	(None, 2121, 16)	0	batch_normalization_8[0][0] batch_normalization_5[0][0]
**activation_8 (Activation)**	(None, 2121, 16)	0	add_2[0][0]
**conv1d_9 (Conv1D)**	(None, 1061, 32)	1568	activation_8[0][0]
**batch_normalization_9 (Batch_normalization)**	(None, 1061, 32)	128	conv1d_9[0][0]
**activation_9 (Activation)**	(None, 1061, 32)	0	batch_normalization_9[0][0]
**conv1d_10 (Conv1D)**	(None, 1061, 32)	3104	activation_9[0][0]
**conv1d_11 (Conv1D)**	(None, 1061, 32)	3104	conv1d_10[0][0]
**batch_normalization_10 (Batch_normalization)**	(None, 1061, 32)	128	conv1d_11[0][0]

**add_3 (Add)**	(None, 1061, 32)	0	batch_normalization_10[0][0]<br>batch_normalization_9[0][0]
**activation_10 (Activation)**	(None, 1061, 32)	0	add_3[0][0]
**conv1d_12 (Conv1D)**	(None, 1061, 32)	3104	activation_10[0][0]
**batch_normalization_11 (Batch_normalization)**	(None, 1061, 32)	128	conv1d_12[0][0]
**activation_11 (Activation)**	(None, 1061, 32)	0	batch_normalization_11[0][0]
**conv1d_13 (Conv1D)**	(None, 1061, 32)	3104	activation_11[0][0]
**batch_normalization_12 (Batch_normalization)**	(None, 1061, 32)	128	conv1d_13[0][0]
**add_4 (Add)**	(None, 1061, 32)	0	batch_normalization_12[0][0]<br>batch_normalization_11[0][0]
**activation_12 (Activation)**	(None, 1061, 32)	0	add_4[0][0]
**conv1d_14 (Conv1D)**	(None, 1061, 32)	3104	activation_12[0][0]
**batch_normalization_13 (Batch_normalization)**	(None, 1061, 32)	128	conv1d_14[0][0]
**activation_13 (Activation)**	(None, 1061, 32)	0	batch_normalization_13[0][0]
**conv1d_15 (Conv1D)**	(None, 1061, 32)	3104	activation_13[0][0]
**batch_normalization_14 (Batch_normalization)**	(None, 1061, 32)	128	conv1d_15[0][0]
**add_5 (Add)**	(None, 1061, 32)	0	batch_normalization_14[0][0]<br>batch_normalization_13[0][0]
**activation_14 (Activation)**	(None, 1061, 32)	0	add_5[0][0]
**conv1d_16 (Conv1D)**	(None, 531, 64)	6208	activation_14[0][0]
**batch_normalization_15 (Batch_normalization)**	(None, 531, 64)	256	conv1d_16[0][0]

**activation_15 (Activation)**	(None, 531, 64)	0	batch_normalization_15[0][0]
**conv1d_17 (Conv1D)**	(None, 531, 64)	12352	activation_15[0][0]
**conv1d_18 (Conv1D)**	(None, 531, 64)	12352	conv1d_17[0][0]
**batch_normalization_16 (Batch_normalization)**	(None, 531, 64)	256	conv1d_18[0][0]
**add_6 (Add)**	(None, 531, 64)	0	batch_normalization_16[0][0] batch_normalization_15[0][0]
**activation_16 (Activation)**	(None, 531, 64)	0	add_6[0][0]
**conv1d_19 (Conv1D)**	(None, 531, 64)	12352	activation_16[0][0]
**batch_normalization_17 (Batch_normalization)**	(None, 531, 64)	256	conv1d_19[0][0]
**activation_17 (Activation)**	(None, 531, 64)	0	batch_normalization_17[0][0]
**conv1d_20 (Conv1D)**	(None, 531, 64)	12352	activation_17[0][0]
**batch_normalization_18 (Batch_normalization)**	(None, 531, 64)	256	conv1d_20[0][0]
**add_7 (Add)**	(None, 531, 64)	0	batch_normalization_18[0][0] batch_normalization_17[0][0]
**activation_18 (Activation)**	(None, 531, 64)	0	add_7[0][0]
**conv1d_21 (Conv1D)**	(None, 531, 64)	12352	activation_18[0][0]
**batch_normalization_19 (Batch_normalization)**	(None, 531, 64)	256	conv1d_21[0][0]
**activation_19 (Activation)**	(None, 531, 64)	0	batch_normalization_19[0][0]
**conv1d_22 (Conv1D)**	(None, 531, 64)	12352	activation_19[0][0]
**batch_normalization_20 (Batch_normalization)**	(None, 531, 64)	256	conv1d_22[0][0]

**add_8 (Add)**	(None, 531, 64)	0	batch_normalization_20[0][0]<br>batch_normalization_19[0][0]
**activation_20 (Activation)**	(None, 531, 64)	0	add_8[0][0]
**flatten_1 (Flatten)**	(None, 33984)	0	activation_20[0][0]
**dense1 (Dense)**	(None, 2048)	69601280	flatten_1[0][0]
**batch_normalization_21 (Batch_normalization)**	(None, 2048)	8192	dense1[0][0]
**dropout1 (Dropout)**	(None, 2048)	0	batch_normalization_21[0][0]
**activation_21 (Activation)**	(None, 2048)	0	dropout1[0][0]
**dense5 (Dense)**	(None, 1024)	2098176	activation_21[0][0]
**batch_normalization_22 (Batch_normalization)**	(None, 1024)	4096	dense5[0][0]
**dropout5 (Dropout)**	(None, 1024)	0	batch_normalization_22[0][0]
**activation_22 (Activation)**	(None, 1024)	0	dropout5[0][0]
**dense6 (Dense)**	(None, 512)	524800	activation_22[0][0]
**batch_normalization_23 (Batch_normalization)**	(None, 512)	2048	dense6[0][0]
**dropout6 (Dropout)**	(None, 512)	0	batch_normalization_23[0][0]
**activation_23 (Activation)**	(None, 512)	0	dropout6[0][0]
**dense7 (Dense)**	(None, 1024)	525312	activation_23[0][0]
**batch_normalization_24 (Batch_normalization)**	(None, 1024)	4096	dense7[0][0]
**dropout7 (Dropout)**	(None, 1024)	0	batch_normalization_24[0][0]

**add_9 (Add)**	(None, 1024)	0	dropout7[0][0]<br>batch_normalization_22[0][0]
**activation_24 (Activation)**	(None, 1024)	0	add_9[0][0]
**dense8 (Dense)**	(None, 512)	524800	activation_24[0][0]
**batch_normalization_25 (Batch_normalization)**	(None, 512)	2048	dense8[0][0]
**dropout8 (Dropout)**	(None, 512)	0	batch_normalization_25[0][0]
**activation_25 (Activation)**	(None, 512)	0	dropout8[0][0]
**dense9 (Dense)**	(None, 256)	131328	activation_25[0][0]
**batch_normalization_26 (Batch_normalization)**	(None, 256)	1024	dense9[0][0]
**dropout9 (Dropout)**	(None, 256)	0	batch_normalization_26[0][0]
**activation_26 (Activation)**	(None, 256)	0	dropout9[0][0]
**dense10 (Dense)**	(None, 128)	32896	activation_26[0][0]
**batch_normalization_27 (Batch_normalization)**	(None, 128)	512	dense10[0][0]
**dropout10 (Dropout)**	(None, 128)	0	batch_normalization_27[0][0]
**activation_27 (Activation)**	(None, 128)	0	dropout10[0][0]
**predictions (Dense)**	(None, 1)	129	activation_27[0][0]
**Total params: 73,569,617**<br>**Trainable params: 73,557,201**<br>**Non-trainable params: 12,416**			

Supplementary Table S24. Methods and respective parameters for the models for 24 individual drugs in setting 1.

Methods	Parameter options in Keras and scikit-learn
CNN	Total params: 187,306<br>Total number of layers: 13 layers<br>Architecture: Supplementary Table S20, Figure S21<br>Loss function: mean square error (MSE)<br>Optimizer: Adam<br>Learning rate: 0.0002<br>Training epoch: 40<br>Batch size: 20
ResNet	Total params: 66,229,585<br>Total number of layers: 30 layers (including 9-times skip connections)<br>Architecture: Supplementary Table S22, Figure S22<br>Loss function: mean square error (MSE)<br>Optimizer: Adam<br>Learning rate: 0.0002<br>Training epoch: 40<br>Batch size: 20
Lasso	Alpha = 0.001
Ridge	Alpha = 0.001
ElasticNet	Alpha = 0.001
SVR	C = 0.01
XGBoost	Default
Random forest	Default

Supplementary Table S25. Methods and their parameters of models for 24 individual drugs in setting 2.

Methods	Parameter options in Keras and scikit-learn packages
CNN	Total params: 199,306<br>Total number of layers: 13 layers<br>Architecture: Supplementary Table S21, Figure S21<br>Loss function: mean square error (MSE)<br>Optimizer: Adam<br>Learning rate: 0.0002<br>Training epoch: 40<br>Batch size: 20
ResNet	Total params: 73,569,617<br>Total number of layers: 30 layers (including 9-times skip connections)<br>Architecture: Supplementary Table S23, Figure S22<br>Loss function: mean square error (MSE)<br>Optimizer: Adam<br>Learning rate: 0.0002<br>Training epoch: 40<br>Batch size: 20
Lasso	Alpha = 0.001
Ridge	Alpha = 0.001
ElasticNet	Alpha = 0.001
SVR	C = 0.01
XGBoost	Default
Random forest	Default

Supplementary Figure S1. Data structures for setting 1 (24 individual drugs from EC-11K) and setting 2 (24 individual drugs from MC-9K). (a) Settings 1 and **(b)** 2 were used for the one-drug one-model approach of each 24 drugs. Column ln(IC_{50})s represents the output variable for each drug treatment in each cell line.

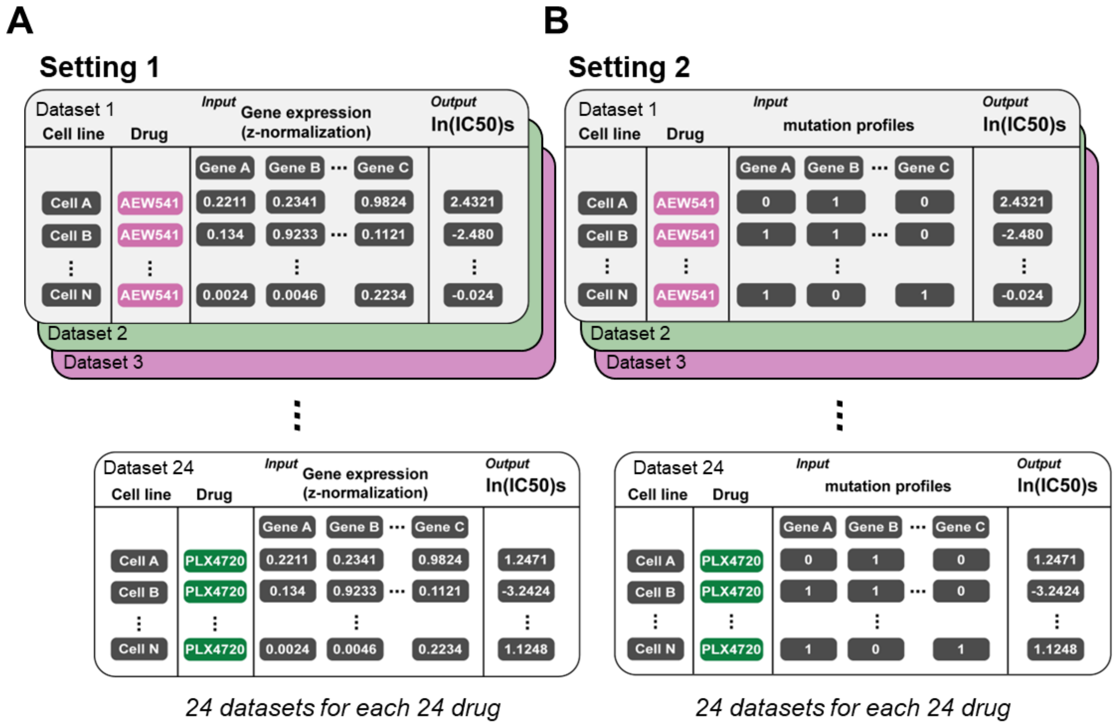

Supplementary Figure S2. The 24 lasso models for 24 individual drugs in setting 1. Considering a drug, the subset from the EC-11K training set was obtained to train the drug's IC_{50} prediction lasso model, which was validated in the subset corresponding to the drug from the EC-11K test set.

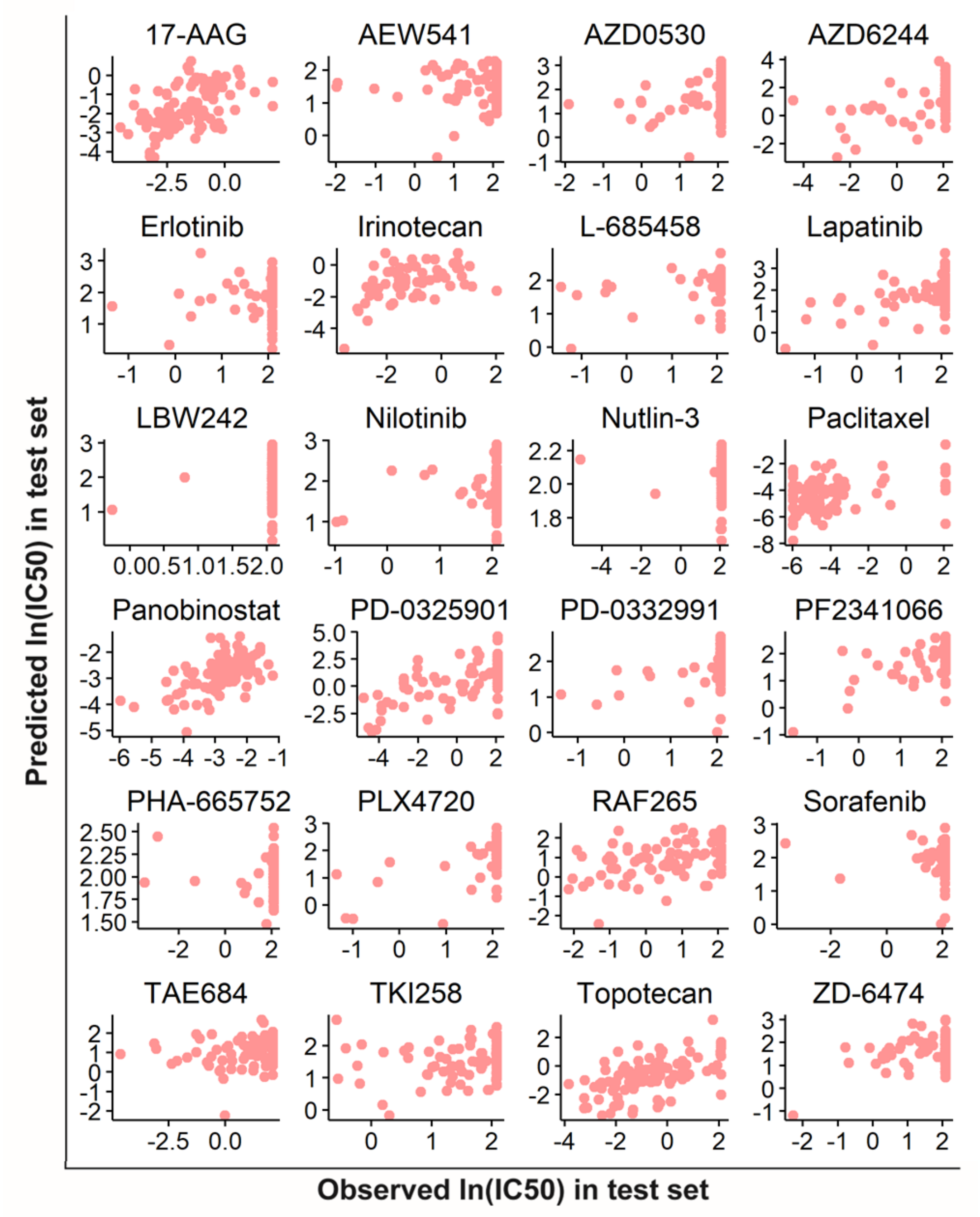

Supplementary Figure S3. The 24 ridge models for 24 individual drugs in setting 1. For each drug, the IC_{50} prediction ridge model was constructed from the EC-11K training set and was validated using the EC-11K test set.

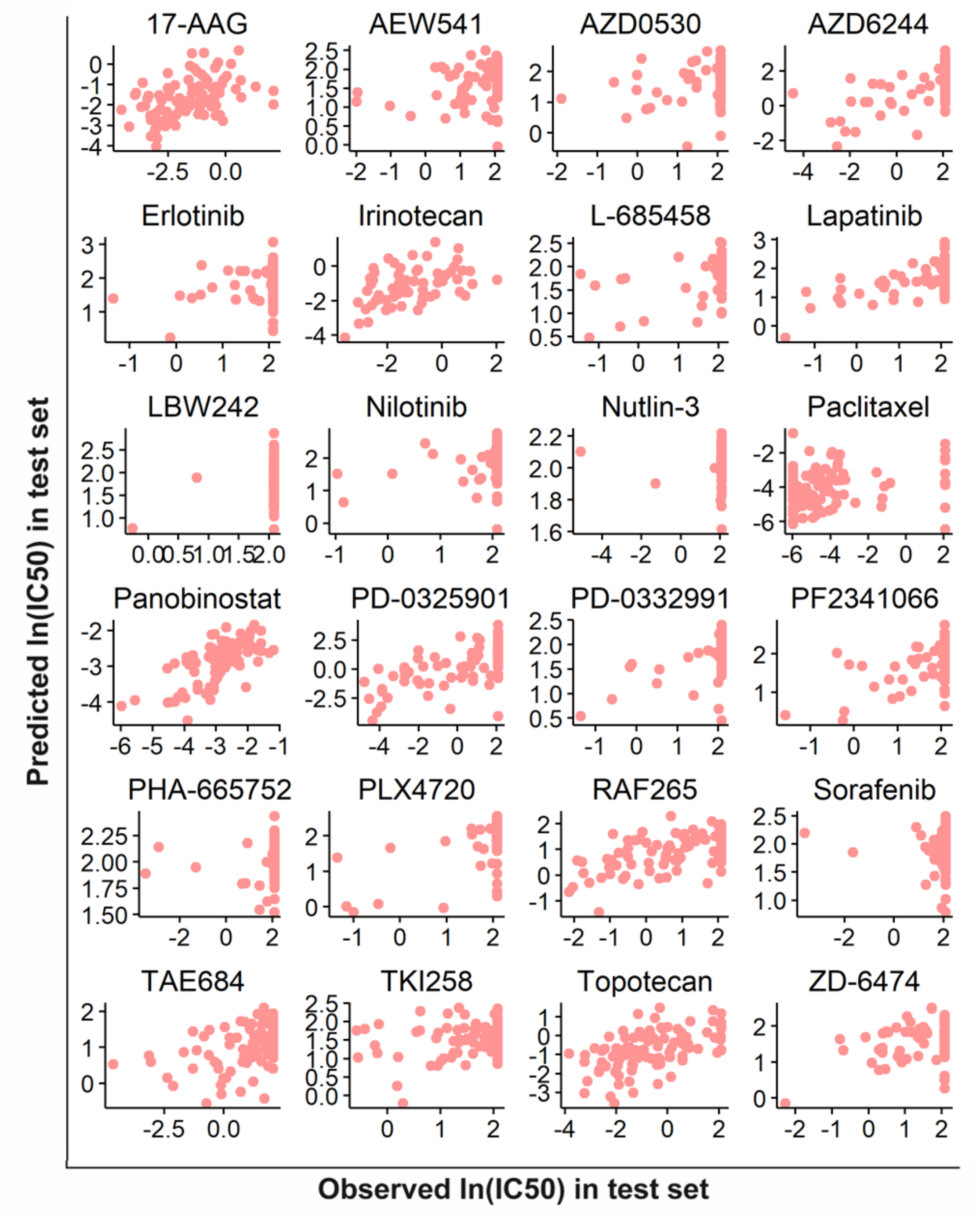

Supplementary Figure S4. The 24 SVR models for 24 individual drugs in setting 1. For each drug, the IC_{50} prediction SVR model was constructed from the EC-11K training set and was validated using the EC-11K test set.

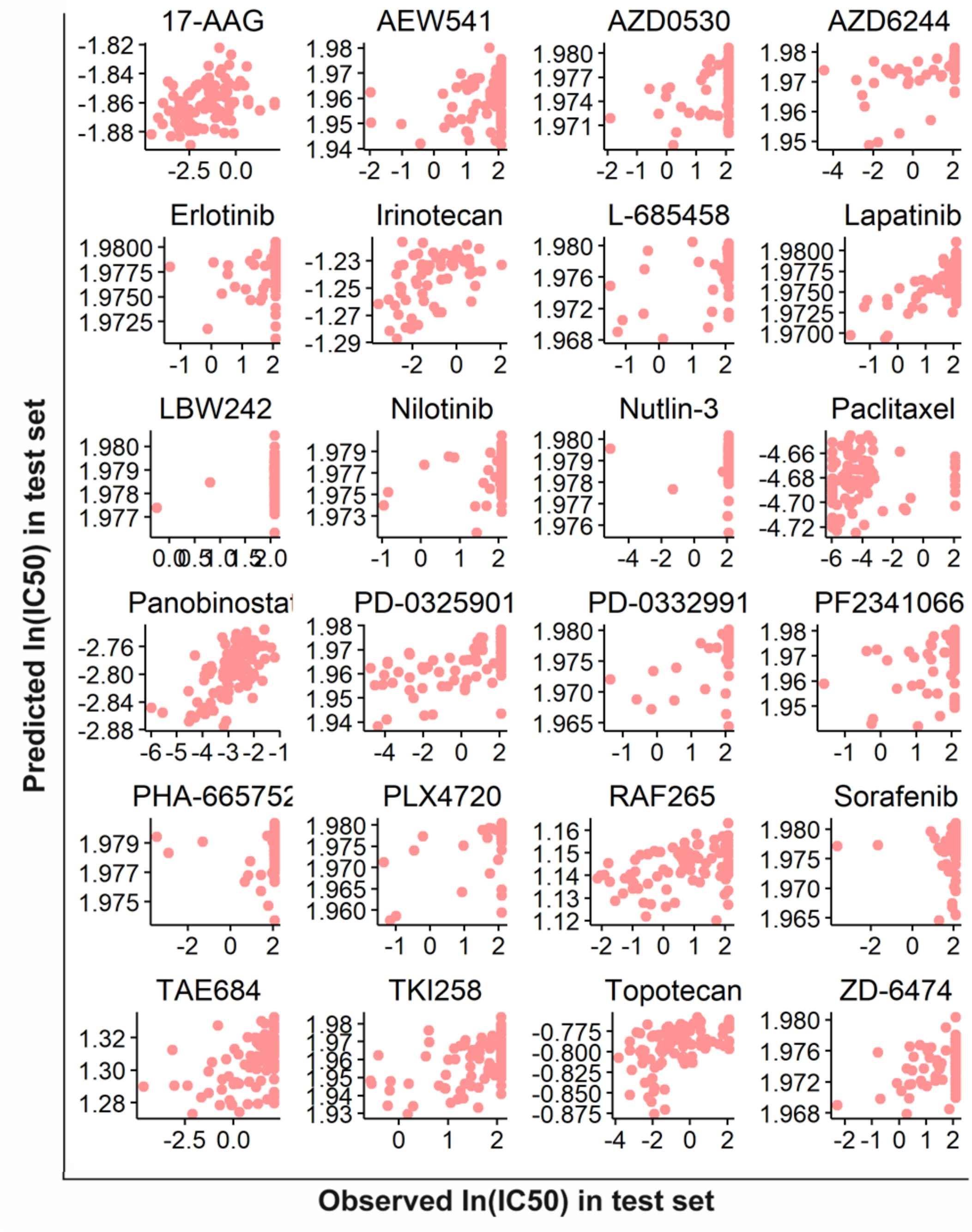

Supplementary Figure S5. The 24 random forest models for 24 individual drugs in setting 1. For each drug, the IC_{50} prediction random forest model was constructed from the EC-11K training set and was validated using the EC-11K test set.

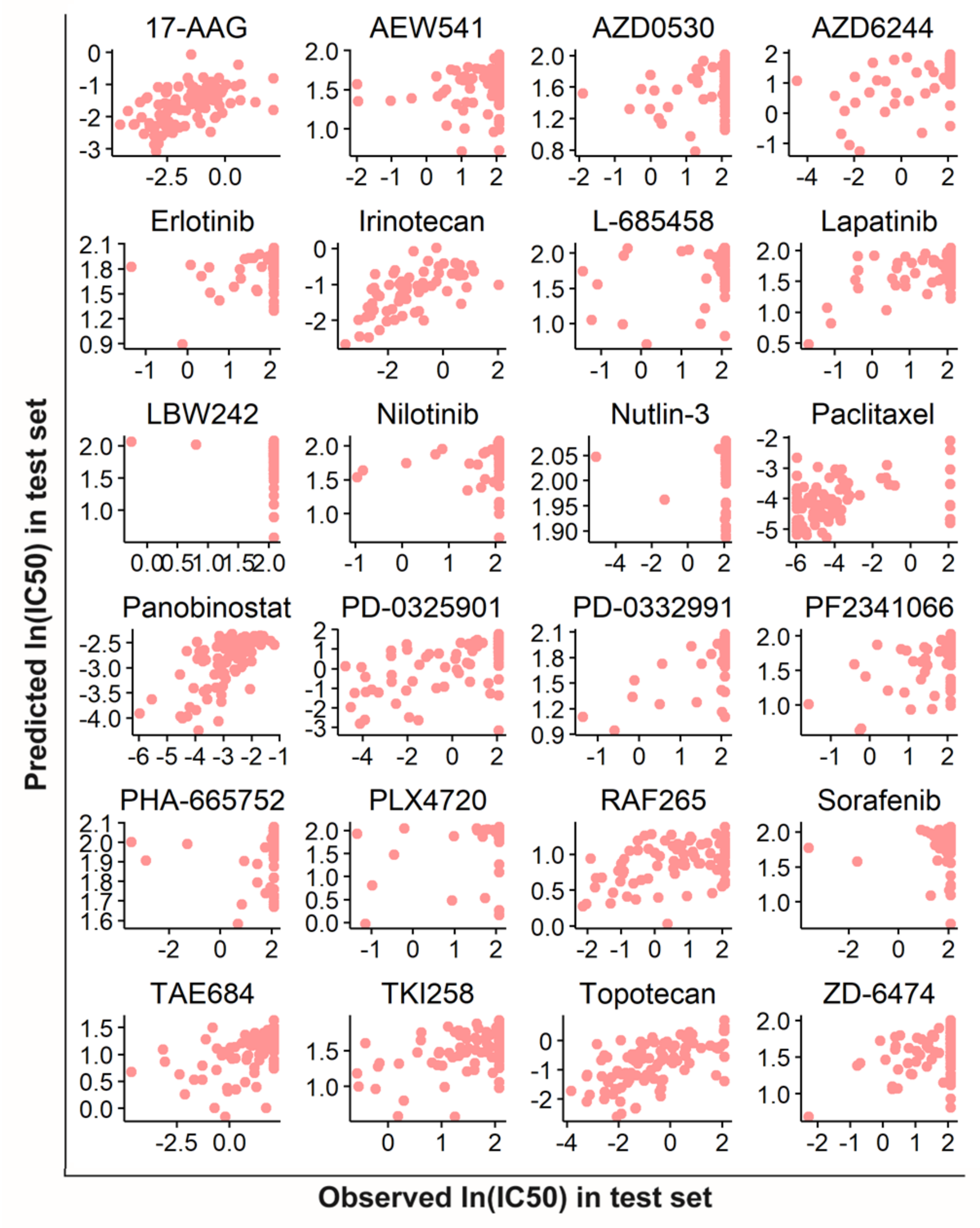

Supplementary Figure S6. The 24 XGBoost models for 24 individual drugs in setting 1. For each drug, the IC_{50} prediction XGBoost model was constructed from the EC-11K training set and was validated using the EC-11K test set.

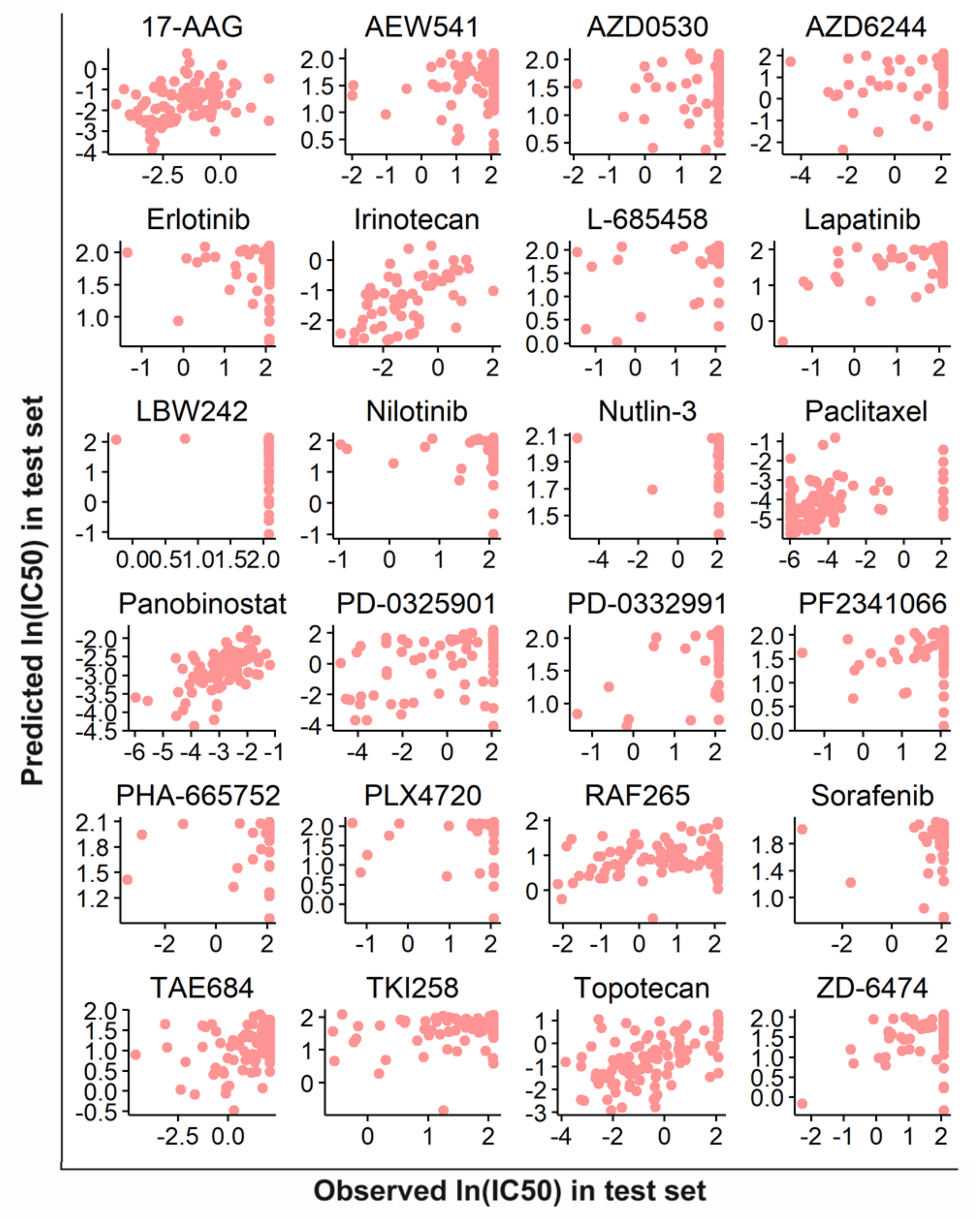

Supplementary Figure S7. The 24 ElasticNet models for 24 individual drugs in setting 1. For each drug, the IC_{50} prediction ridge model was constructed from the EC-11K training set and was validated using the EC-11K test set.

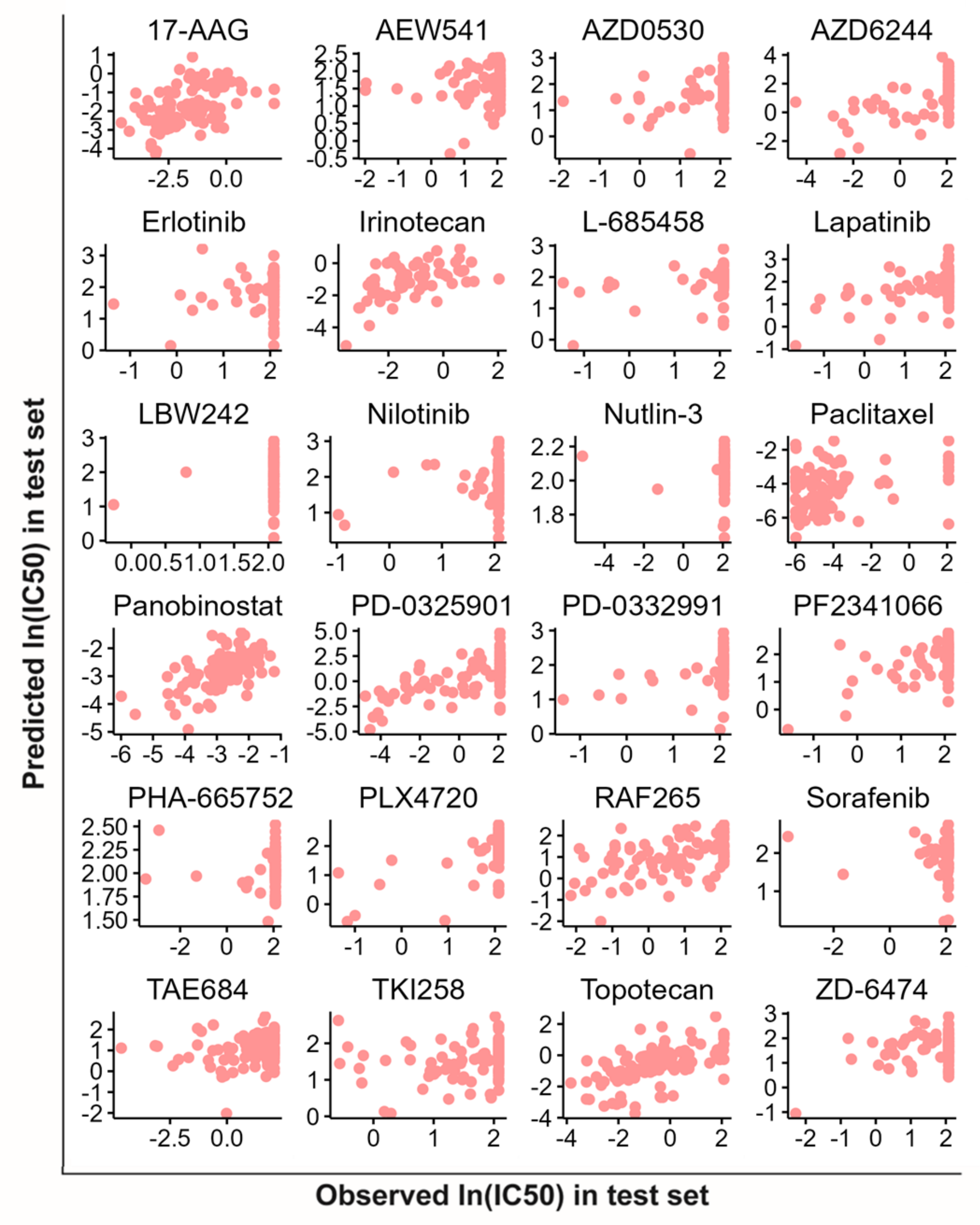

Supplementary Figure S8. The 24 ResNet models for 24 individual drugs in setting 1. For each drug, the IC_{50} prediction ResNet model was constructed from the EC-11K training set and was validated using the EC-11K test set.

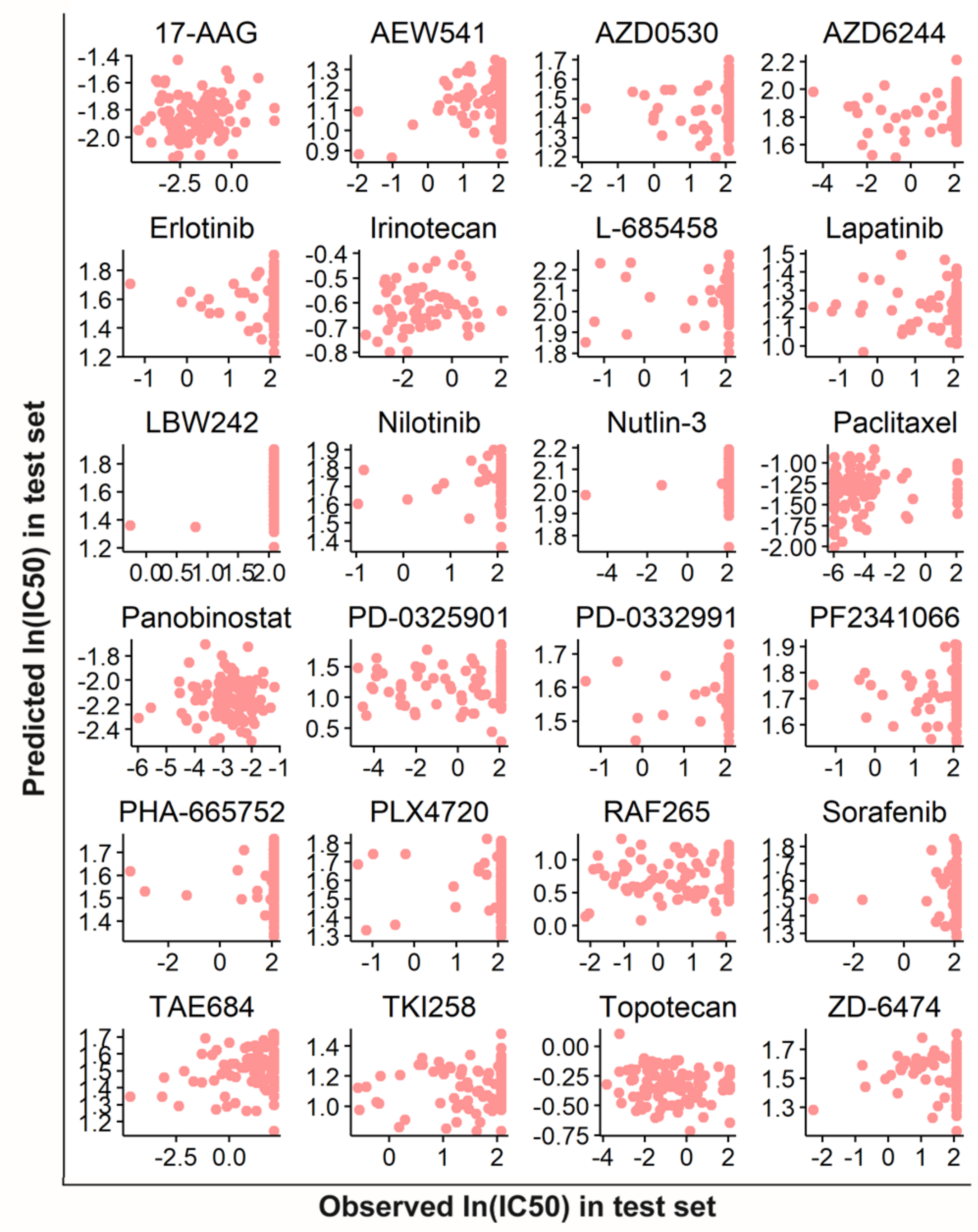

Supplementary Figure S9. The 24 CNN models for 24 individual drugs in setting 1. For each drug, the IC_{50} prediction CNN model was constructed from the EC-11K training set and was validated using the EC-11K test set.

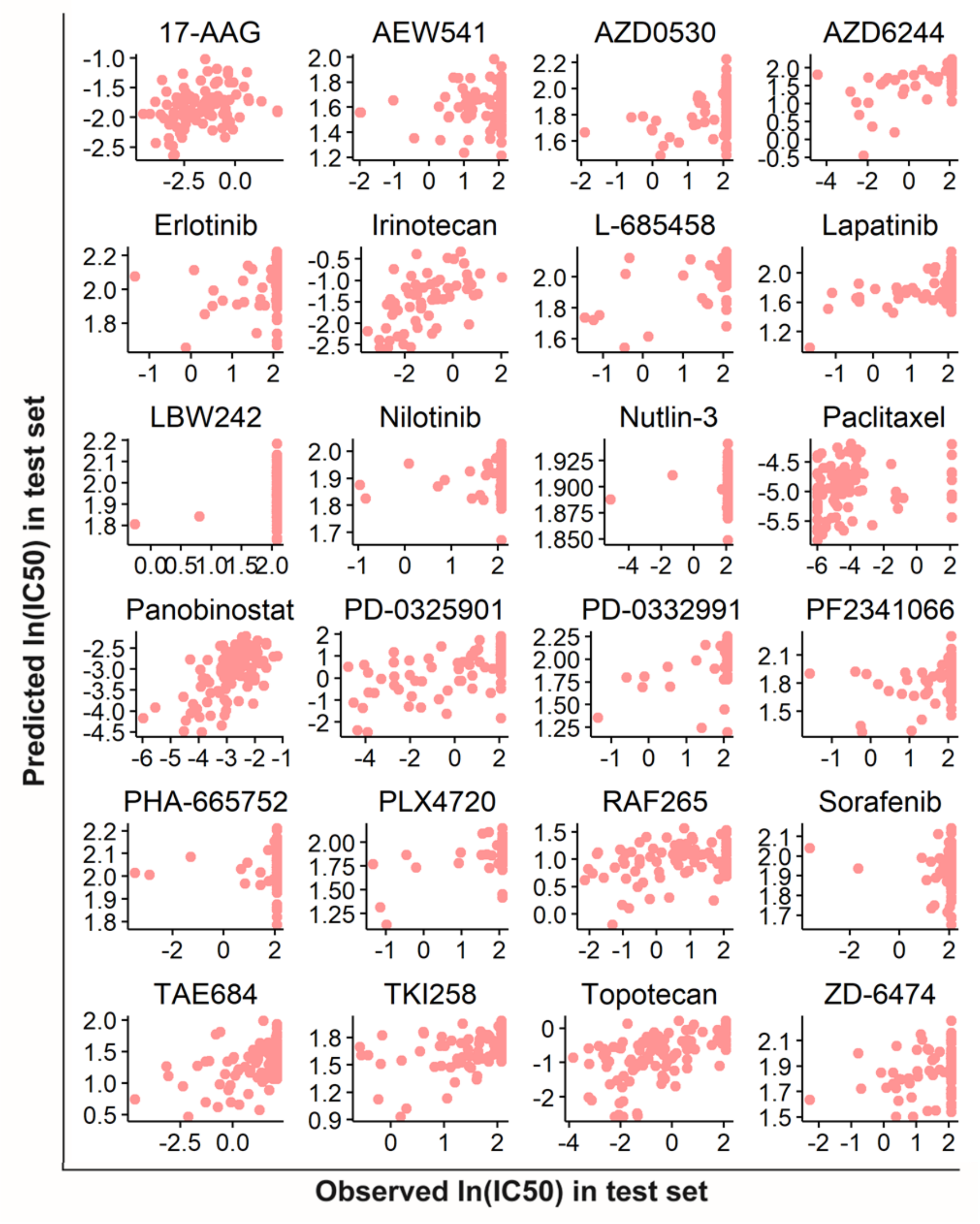

Supplementary Figure S10. Feature selection result of the ridge (panobinostat) model and CNN model. Performances of the panobinostat ridge model and CNN model according to the number of selected features.

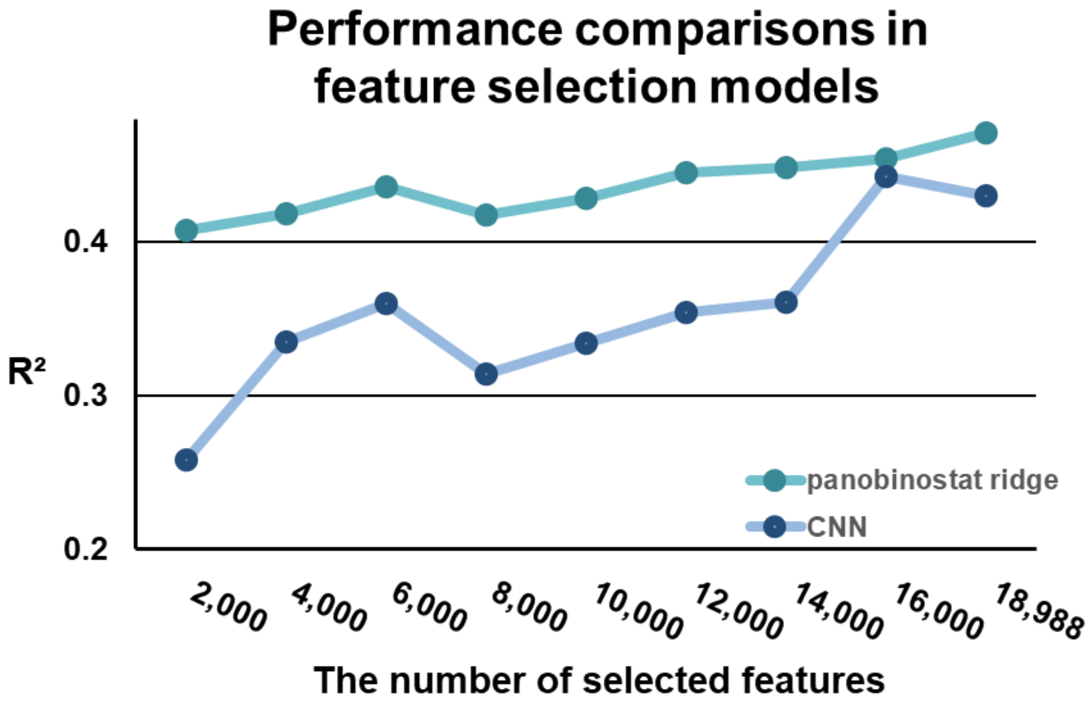

Supplementary Figure S11. The 24 lasso models for 24 individual drugs in setting 2. For each drug, the IC_{50} prediction model was constructed using the MC-9K training set and was validated using the MC-9K test set.

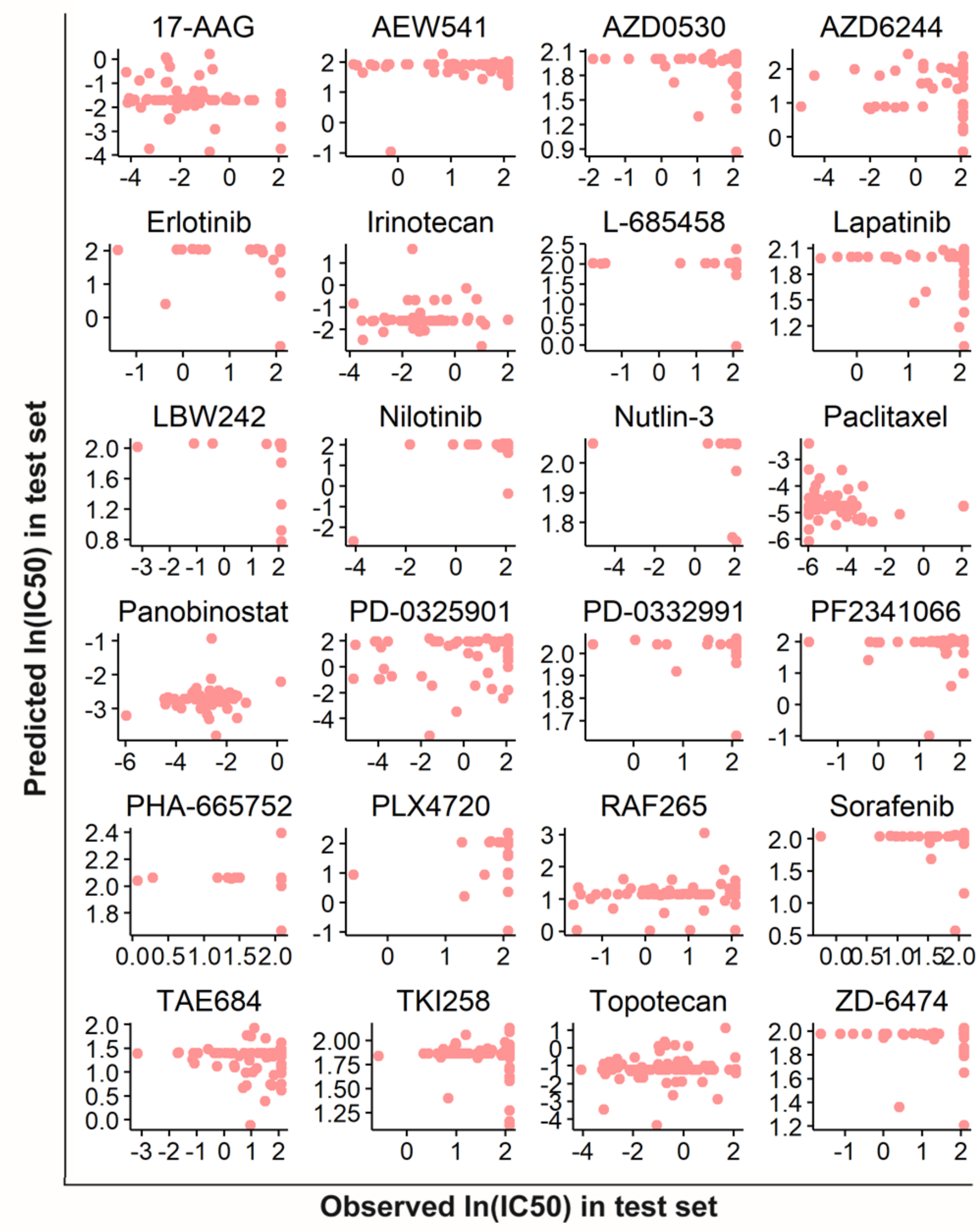

Supplementary Figure S12. The 24 ridge models for 24 individual drugs in setting 2. For each drug, the IC_{50} prediction model was constructed using the MC-9K training set and was validated using the MC-9K test set.

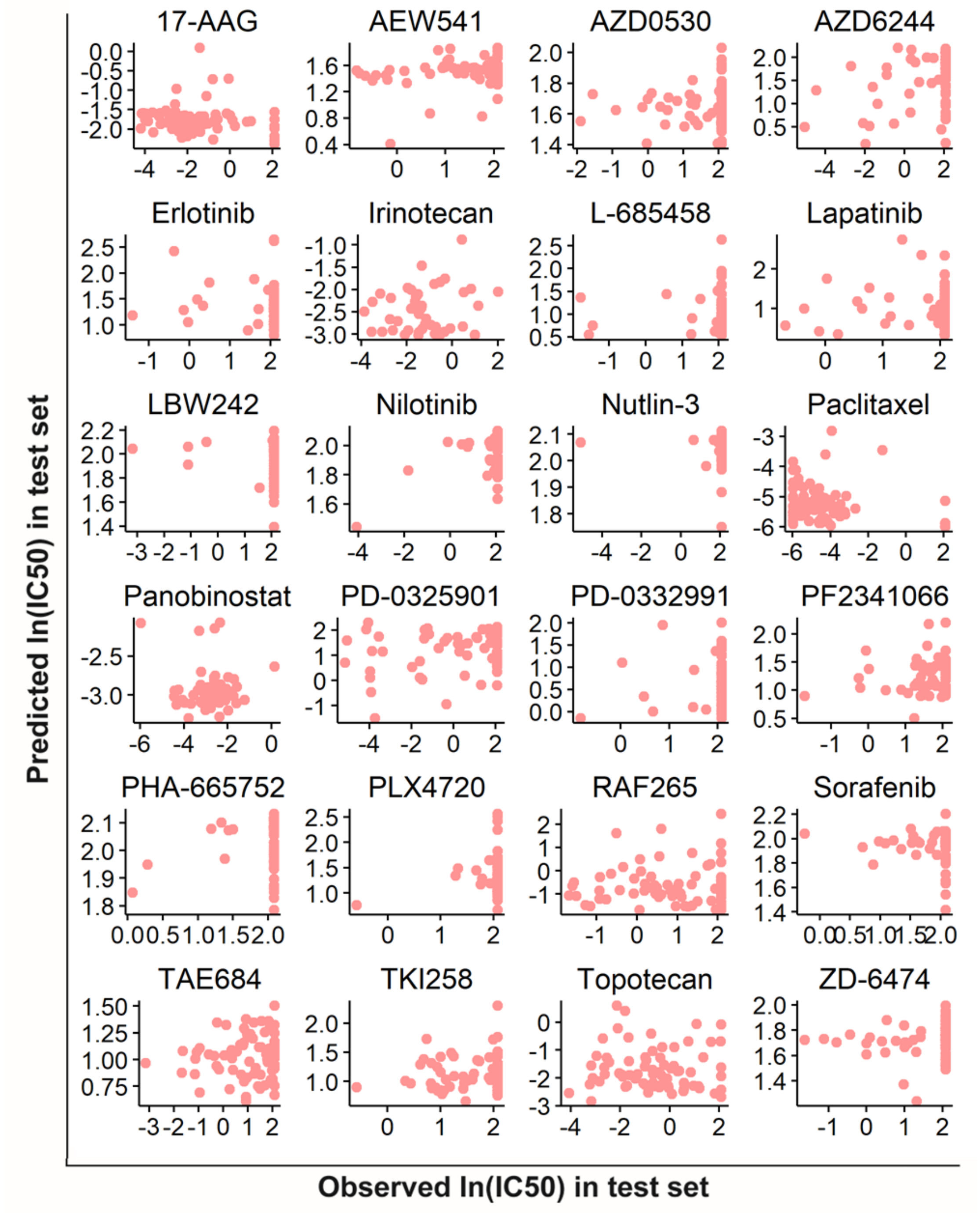

Supplementary Figure S13. The 24 SVR models for 24 individual drugs in setting 2. For each drug, the IC_{50} prediction model was constructed using the MC-9K training set and was validated using the MC-9K test set.

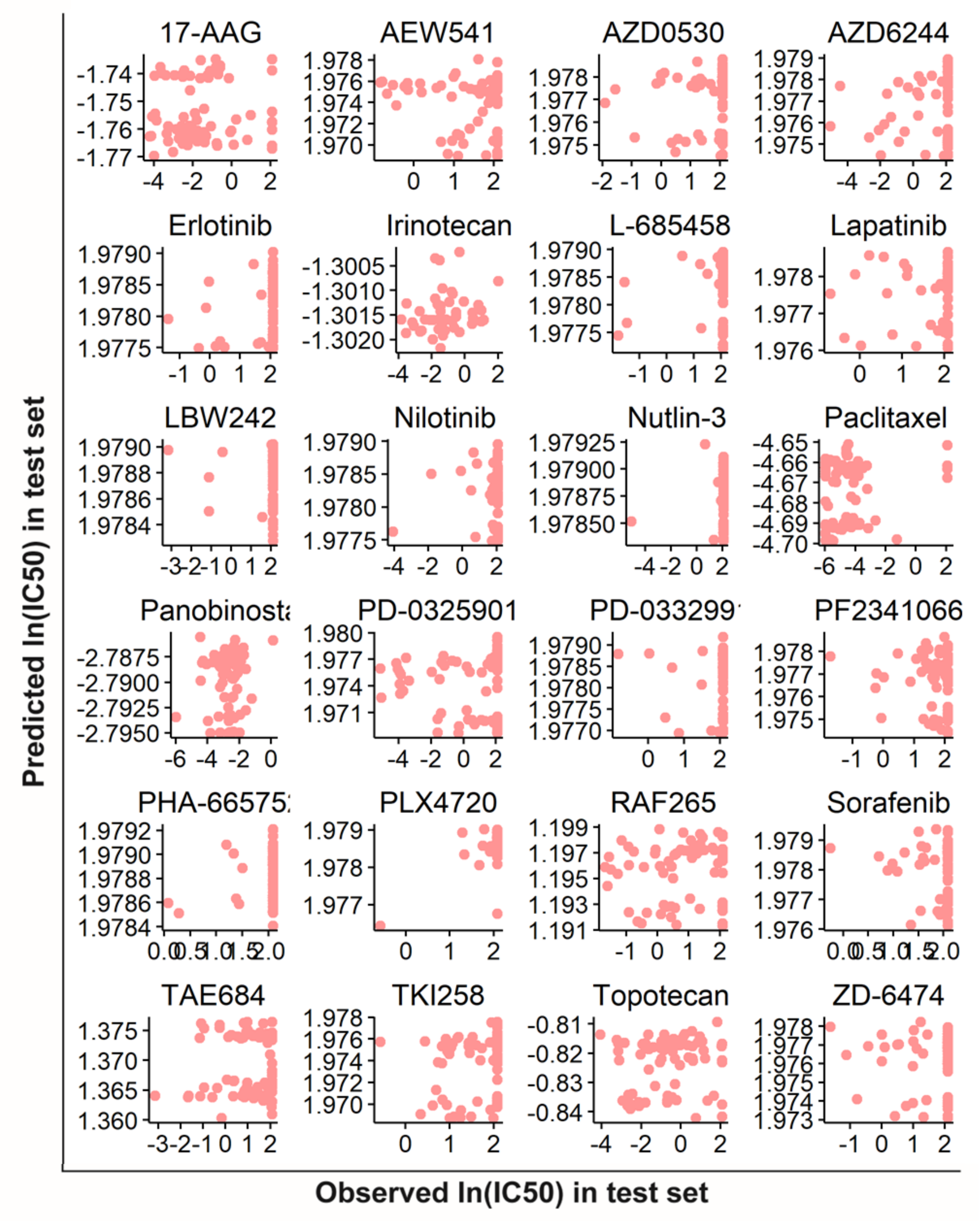

Supplementary Figure S14. The 24 random forest models for 24 individual drugs in setting 2. For each drug, the IC_{50} prediction model was constructed using the MC-9K training set and was validated using the MC-9K test set.

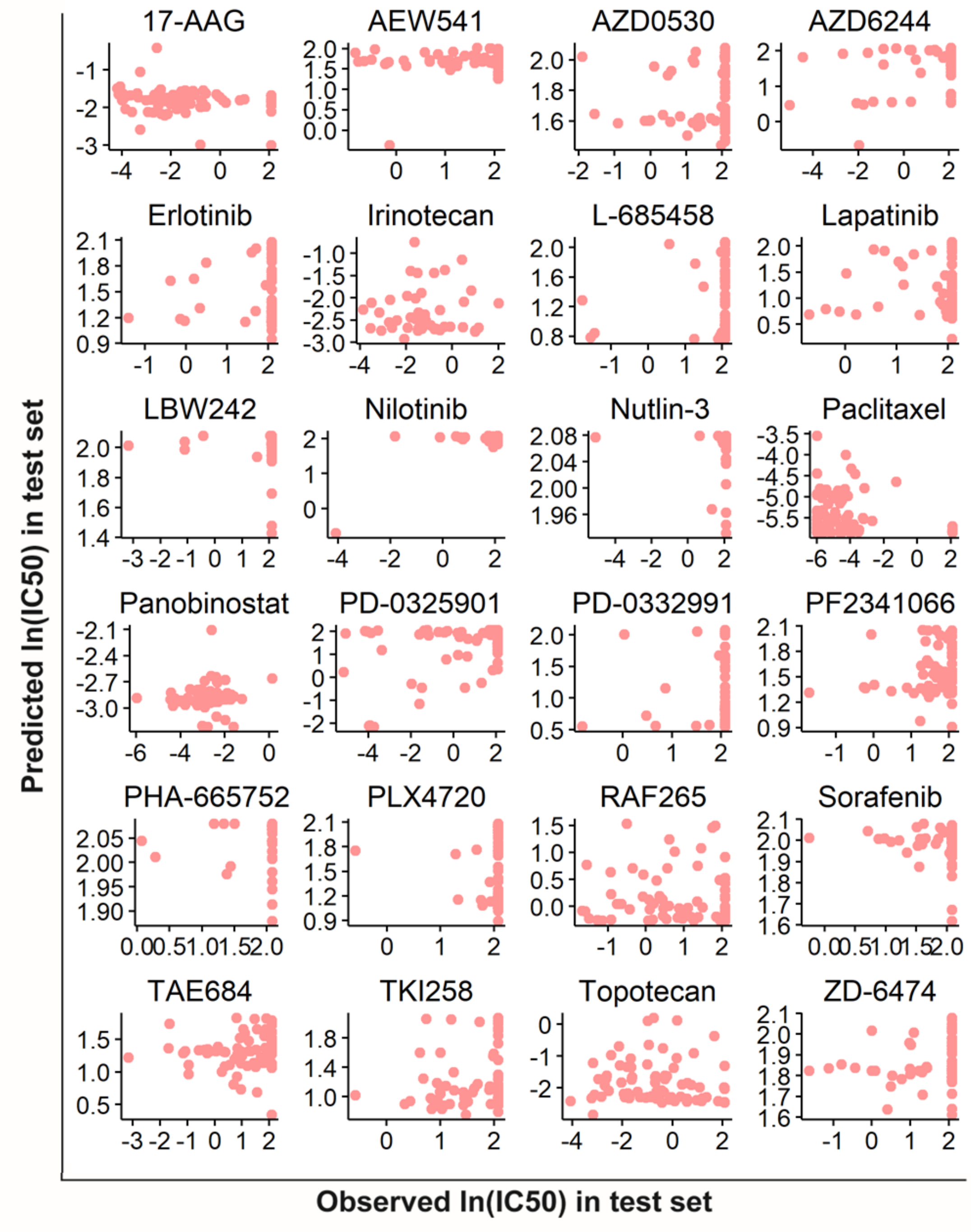

Supplementary Figure S15. The 24 XGBoost models for 24 individual drugs in setting 2. For each drug, the IC_{50} prediction model was constructed using the MC-9K training set and was validated using the MC-9K test set.

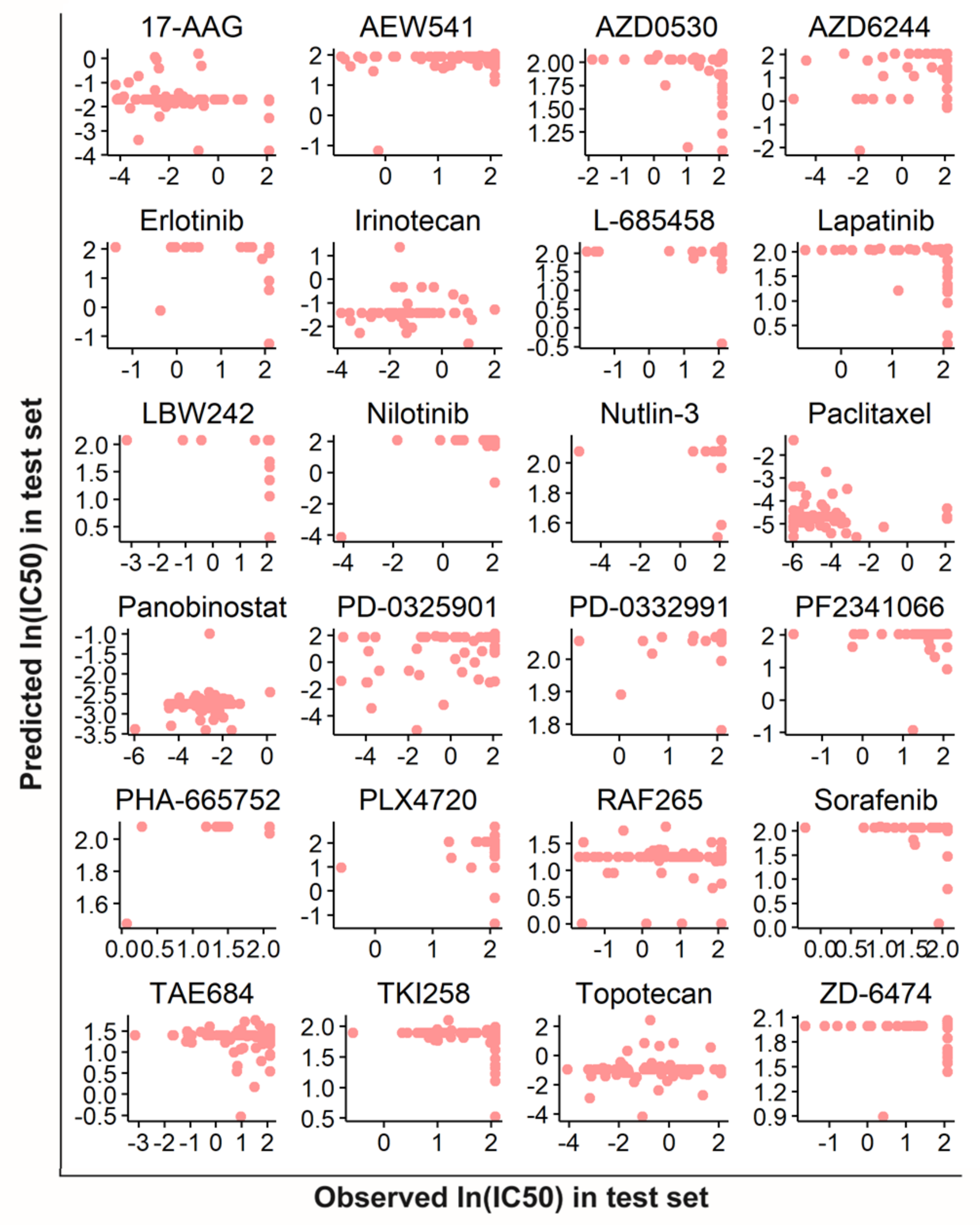

Supplementary Figure S16. The 24 ElasticNet models for 24 individual drugs in setting 2. For each drug, the IC_{50} prediction model was constructed using the MC-9K training set and was validated using the MC-9K test set.

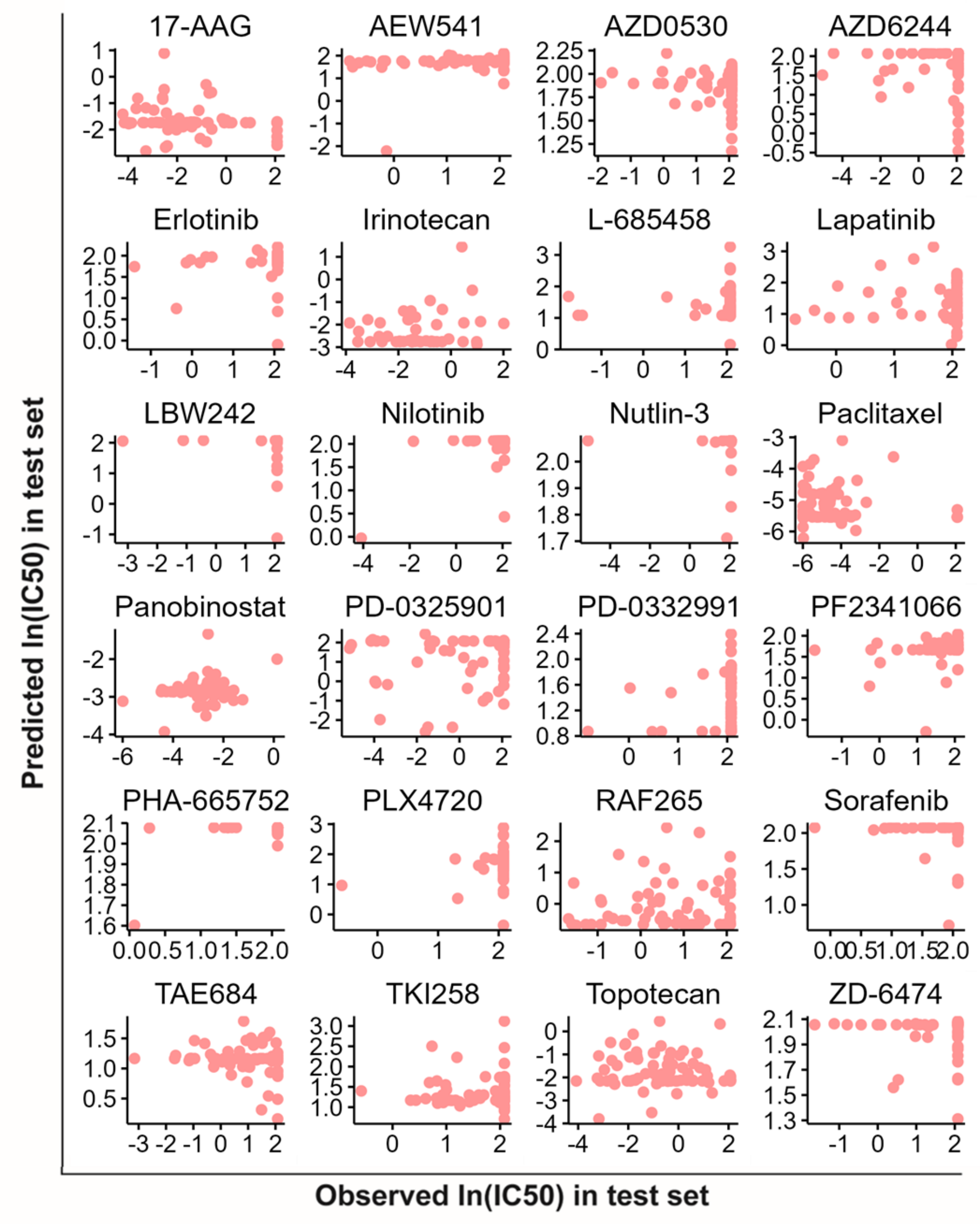

Supplementary Figure S17. The 24 ResNet models for 24 individual drugs in setting 2. For each drug, the IC_{50} prediction model was constructed using the MC-9K training set and was validated using the MC-9K test set.

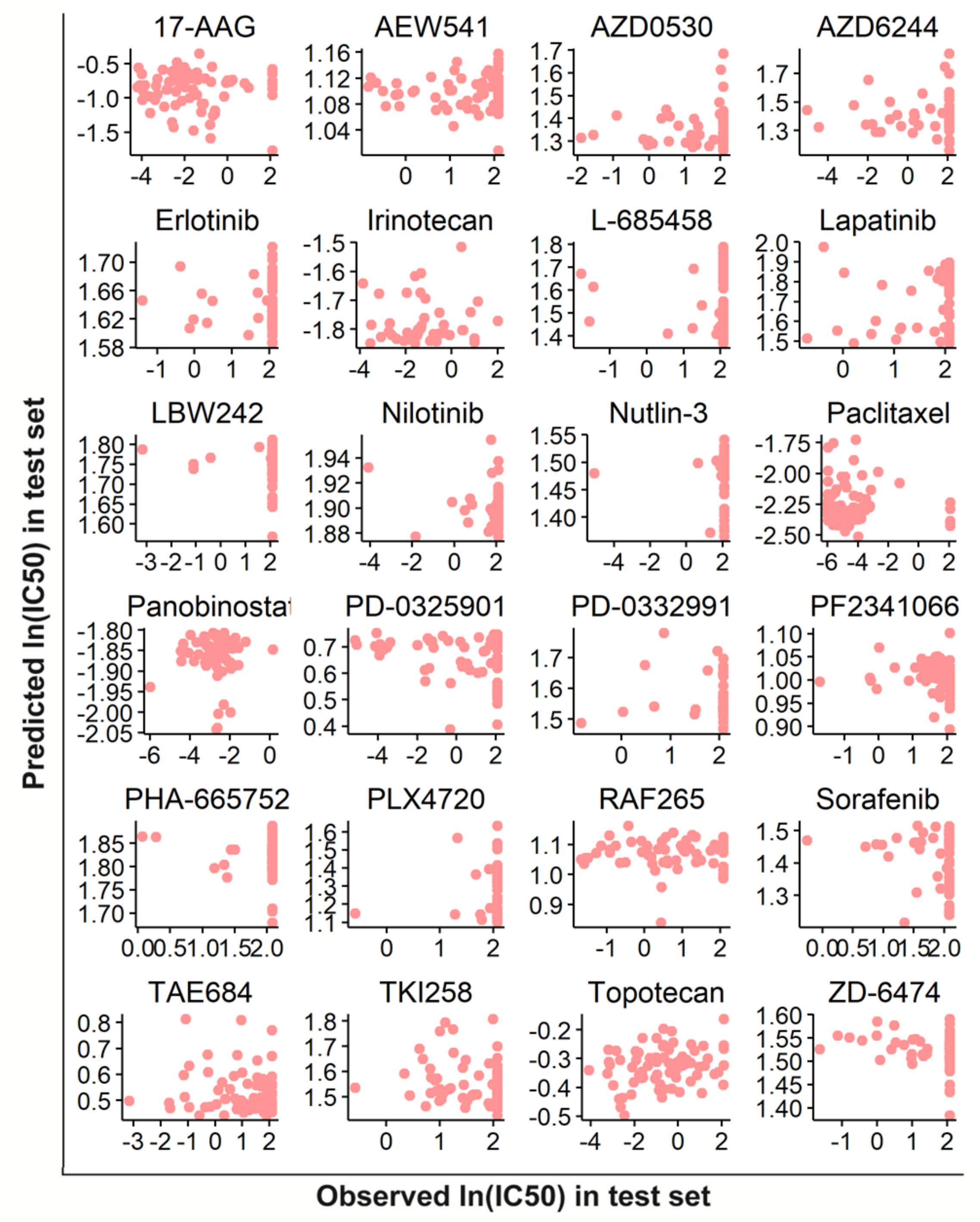

Supplementary Figure S18. The 24 CNN models for 24 individual drugs in setting 2. For each drug, the IC_{50} prediction model was constructed using the MC-9K training set and was validated using the MC-9K test set.

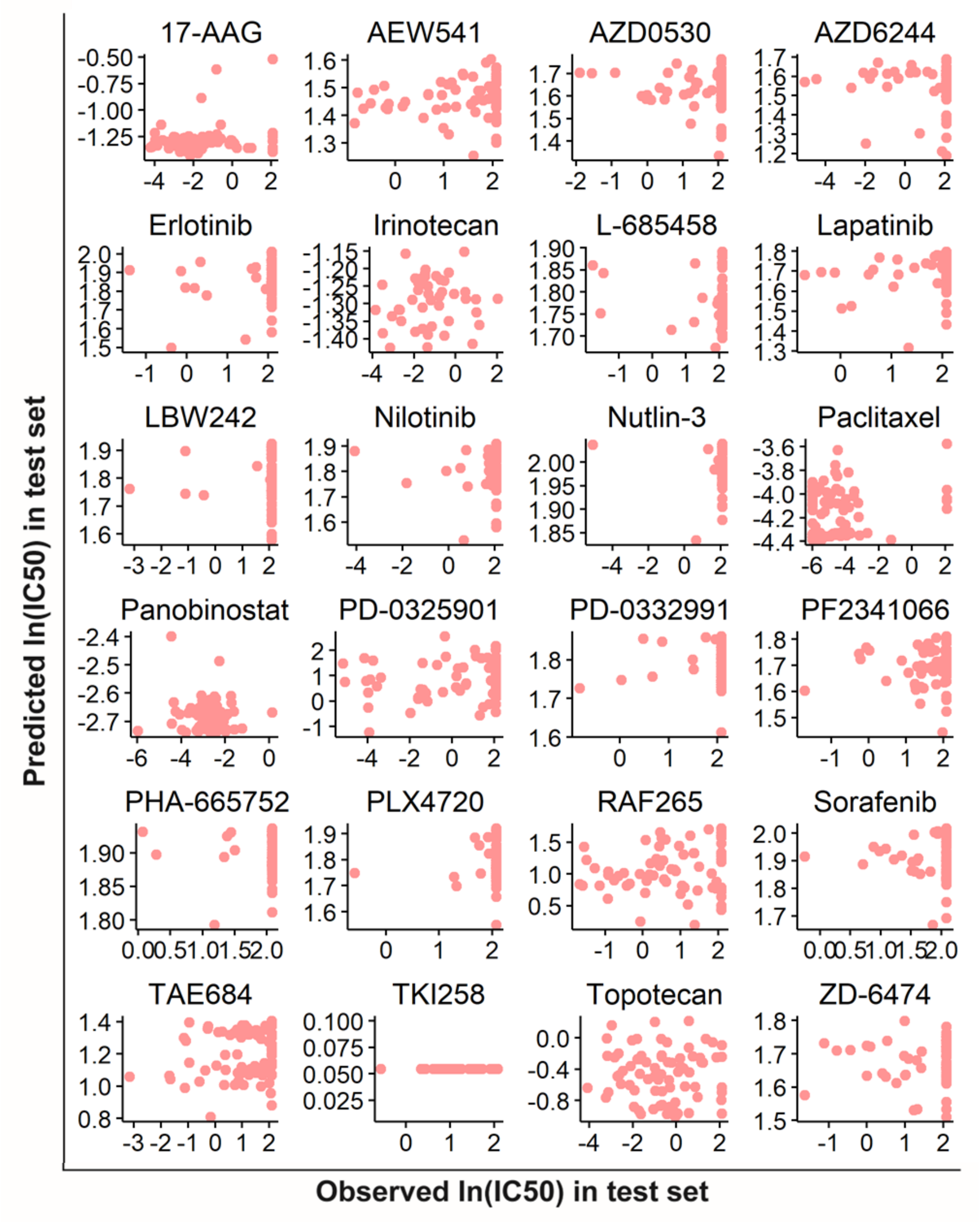

Supplementary Figure S19. Comparison between gene features selected by XAI and those in the ridge (panobinostat) model. For case A through D (Figure 4f through 4i), 100 gene features were selected for each case using XAI, and we obtained 374 non-redundant gene features. We also selected top 100 important gene features from the panobinostat ridge model. We compared the two sets of selected features by the two methods.

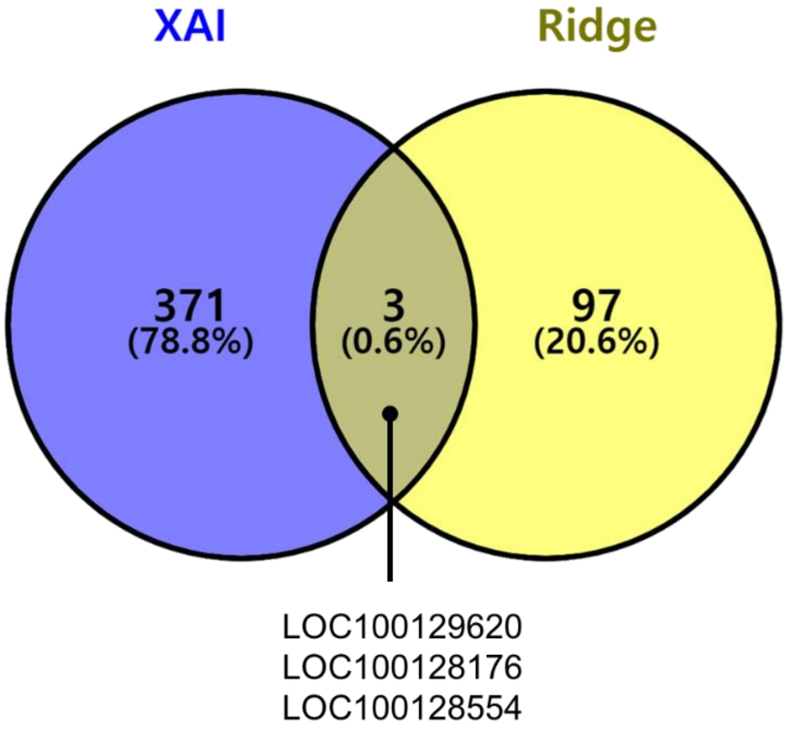

Supplementary Figure S20. Application of XAI analysis to CNN model in the four cases. To identify major genes affecting the CNN model, we performed XAI analysis with the four cases (cases A through D). The six genes affecting drug sensitivity (red bar) and drug resistance (blue bar) were detected in **(a)** case A, **(b)** case B, **(c)** case C, and **(d)** case D, respectively.

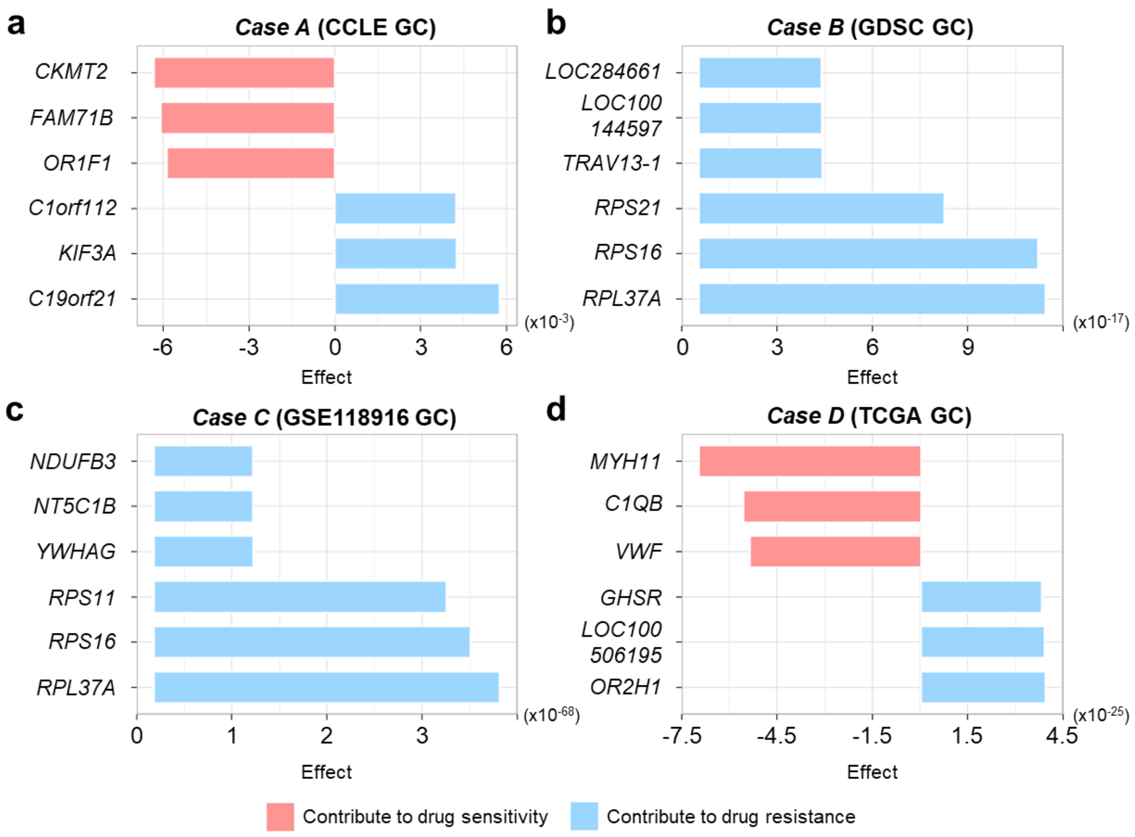

Supplementary Figure S21. CNN structure. Grey boxes represent convolution and fully-connected layers. Cell line genomics vector (e.g., gene expression or mutation) was assigned in the first layer.

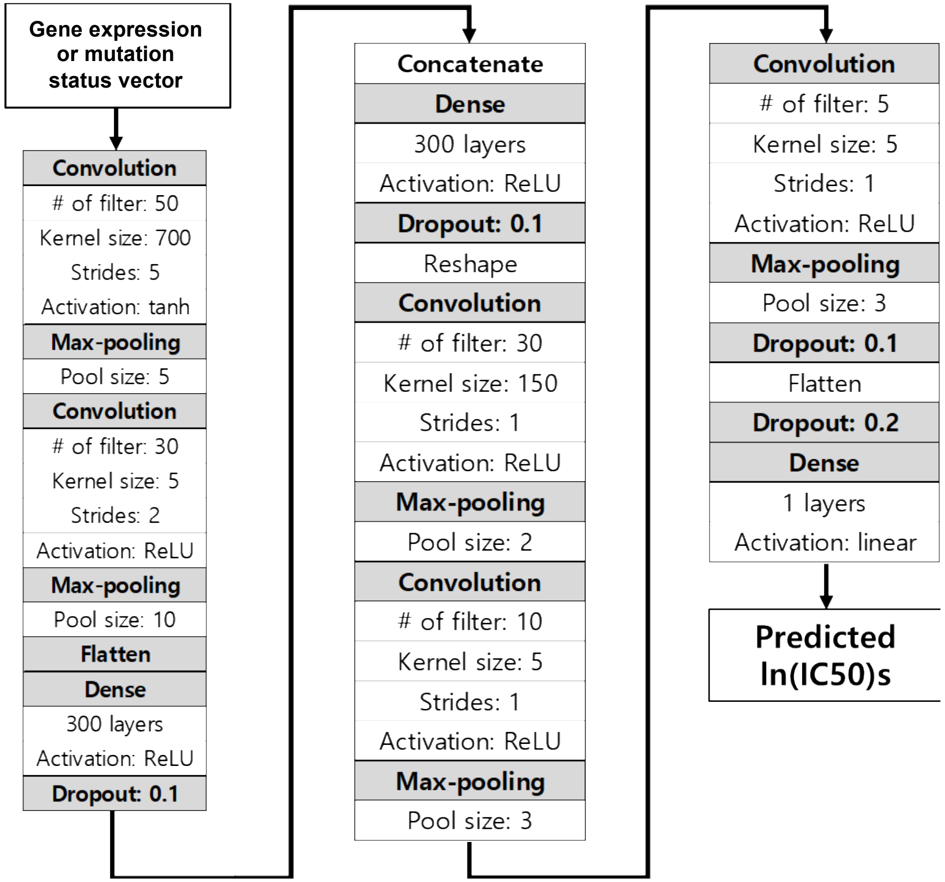

Supplementary Figure S22. ResNet architecture. Grey boxes represent convolution and fully-connected layers. The genomics vector (e.g., gene expression or mutation) was used as the input layer. Skip connections are indicated as blue arrows.

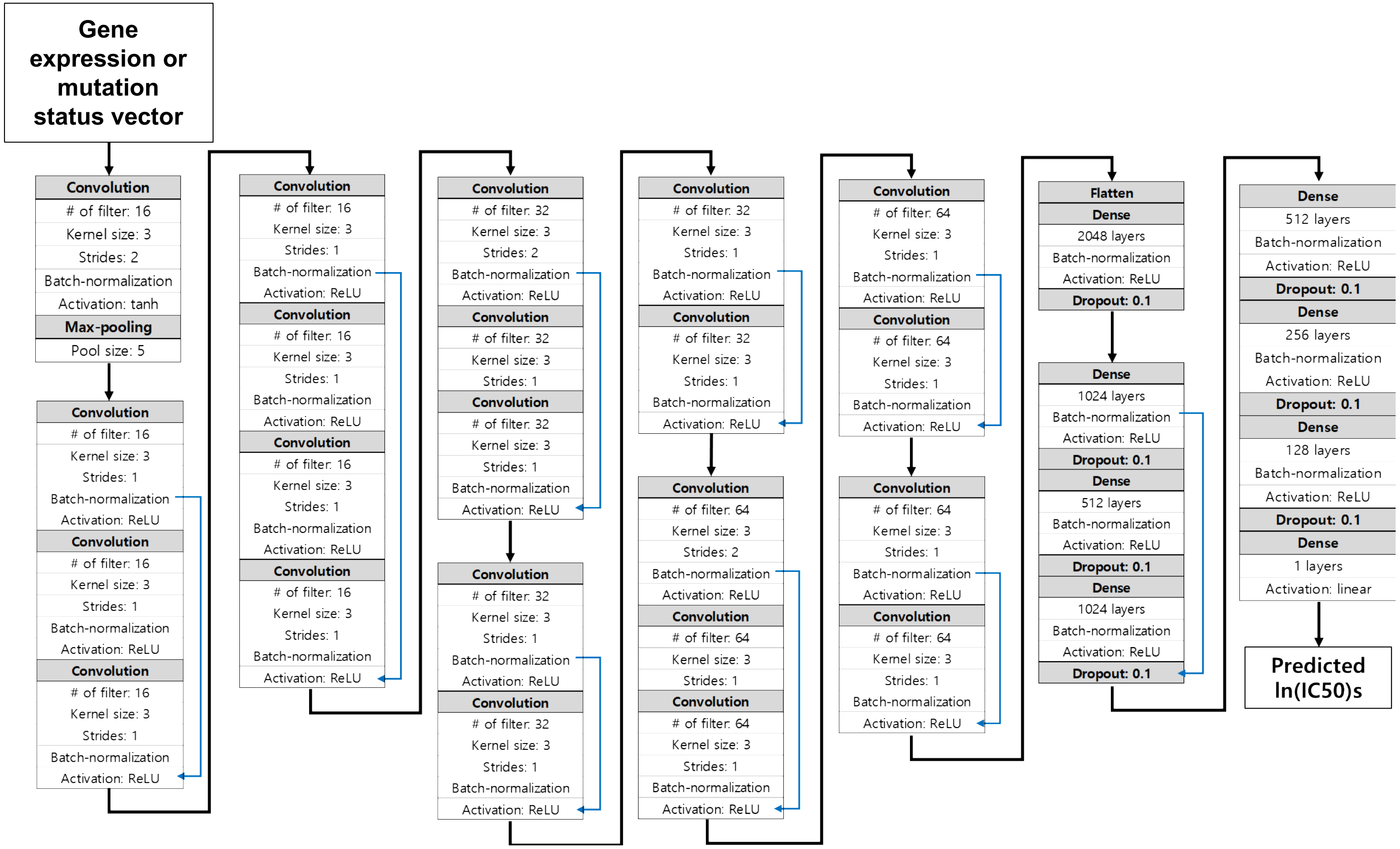

Supplementary References

1 Yang, W. *et al.* Genomics of Drug Sensitivity in Cancer (GDSC): a resource for therapeutic biomarker discovery in cancer cells. *Nucleic acids research* **41**, D955-961, doi:10.1093/nar/gks1111 (2013).

2 Barretina, J. *et al.* The Cancer Cell Line Encyclopedia enables predictive modelling of anticancer drug sensitivity. *Nature* **483**, 603-607, doi:10.1038/nature11003 (2012).
